# Blue light induces a neuroprotective gene expression program in *Drosophila* photoreceptors

**DOI:** 10.1186/s12868-018-0443-y

**Published:** 2018-07-20

**Authors:** Hana Hall, Jingqun Ma, Sudhanshu Shekhar, Walter D. Leon-Salas, Vikki M. Weake

**Affiliations:** 1 Department of Biochemistry, Purdue University, West Lafayette, IN 47907 USA; 2 Present Address: Janelia Research Campus, Ashburn, VA 20147 USA; 3 Interdisciplinary Life Science (PULSe), Purdue University, West Lafayette, IN 47907 USA; 4 Purdue Polytechnic Institute, Purdue University, West Lafayette, IN 47907 USA; 5 Purdue University Center for Cancer Research, Purdue University, West Lafayette, 47907 USA

**Keywords:** *Drosophila*, Blue light, Retinal degeneration, Transcriptome, Photoreceptor, RNA-seq

## Abstract

**Background:**

Light exposure induces oxidative stress, which contributes to ocular diseases of aging. Blue light provides a model for light-induced oxidative stress, lipid peroxidation and retinal degeneration in *Drosophila melanogaster*. In contrast to mature adults, which undergo retinal degeneration when exposed to prolonged blue light, newly-eclosed flies are resistant to blue light-induced retinal degeneration. Here, we sought to characterize the gene expression programs induced by blue light in flies of different ages to identify neuroprotective pathways utilized by photoreceptors to cope with light-induced oxidative stress.

**Results:**

To identify gene expression changes induced by blue light exposure, we profiled the nuclear transcriptome of *Drosophila* photoreceptors from one- and six-day-old flies exposed to blue light and compared these with dark controls. Flies were exposed to 3 h blue light, which increases levels of reactive oxygen species but does not cause retinal degeneration. We identified substantial gene expression changes in response to blue light only in six-day-old flies. In six-day-old flies, blue light induced a neuroprotective gene expression program that included upregulation of stress response pathways and downregulation of genes involved in light response, calcium influx and ion transport. An intact phototransduction pathway and calcium influx were required for upregulation, but not downregulation, of genes in response to blue light, suggesting that distinct pathways mediate the blue light-associated transcriptional response.

**Conclusion:**

Our data demonstrate that under phototoxic conditions, *Drosophila* photoreceptors upregulate stress response pathways and simultaneously, downregulate expression of phototransduction components, ion transporters, and calcium channels. Together, this gene expression program both counteracts the calcium influx resulting from prolonged light exposure, and ameliorates the oxidative stress resulting from this calcium influx. Thus, six-day-old flies can withstand up to 3 h blue light exposure without undergoing retinal degeneration. Developmental transitions during the first week of adult *Drosophila* life lead to an altered gene expression program in photoreceptors that includes reduced expression of genes that maintain redox and calcium homeostasis, reducing the capacity of six-day-old flies to cope with longer periods (8 h) of light exposure. Together, these data provide insight into the neuroprotective gene regulatory mechanisms that enable photoreceptors to withstand light-induced oxidative stress.

**Electronic supplementary material:**

The online version of this article (10.1186/s12868-018-0443-y) contains supplementary material, which is available to authorized users.

## Background

Light itself, although essential for vision, poses a stress to the visual system through photogeneration of reactive oxygen species [1]. Oxidative stress has been linked to the onset of human retinal degeneration [1]. The specialized nature and composition of photoreceptor neurons may increase their sensitivity to oxidative damage due to the energy demands of vision, the high concentration of peroxidation-sensitive polyunsaturated fatty acids, and exposure to light [2, 3]. In particular, lipid peroxidation, the oxidation of membrane lipids, is an emerging hallmark of both neurodegenerative and age-associated ocular disease [3, 4]. Lipid peroxidation, once initiated, induces a cycle of oxidative damage that harms cellular membranes and eventually culminates in cell death [5]. Cells possess endogenous protective mechanisms to withstand lipid peroxidation and maintain redox homeostasis including gene regulatory mechanisms [6]. However, the neuroprotective mechanisms utilized by photoreceptors to withstand the oxidative stress generated as a normal part of light exposure are not fully understood.

In *Drosophila*, as in other organisms, blue light wavelengths induce retinal degeneration [7–9]. Blue light (λ = 480 nm) activates the G-protein coupled receptor Rhodopsin 1 (Rh1) within the rhabdomere, the light sensing organelle, of R1–R6 photoreceptors [10]. Upon blue illumination, Rh1 is activated to metarhodopsin initiating the phototransduction cascade [10]. In flies, metarhodopsin can be converted back to Rh1 by orange light (λ = 580 nm) [10–12]. Persistent production of metarhodopsin in the presence of blue light leads to its endocytosis and prolonged calcium influx, both of which can induce cell death [13–18]. The prolonged calcium influx resulting from blue light exposure increases levels of reactive oxygen species in the eye including hydrogen peroxide and lipid peroxidation [19]. We previously showed that lipid peroxidation is a major contributor to blue light-induced retinal degeneration because feeding flies lipophilic antioxidants, or overexpressing Cytochrome-b5, suppressed lipid peroxidation and enhanced photoreceptor survival [19]. Thus, blue light exposure in flies provides a model for light-induced oxidative stress and lipid peroxidation, hallmarks of age-associated ocular and neurodegenerative disease [3, 4].

**Fig. 1:**
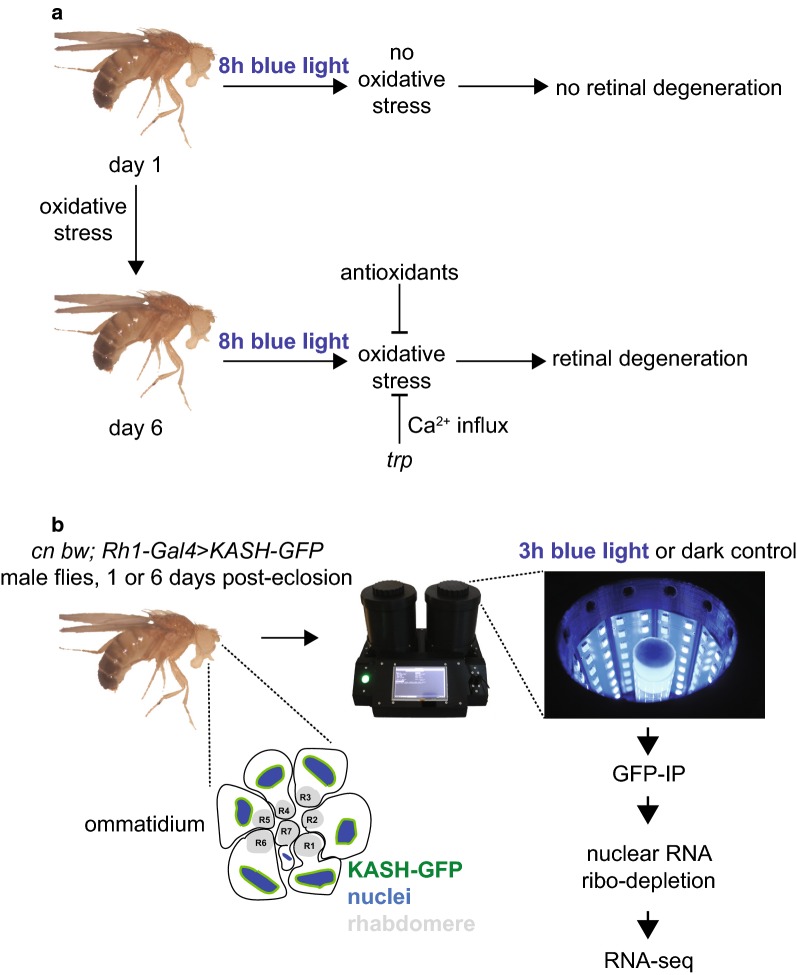
Blue light provides a model for light-induced oxidative stress and retinal degeneration in flies. **a** Six-day-old white-eyed flies undergo retinal degeneration after 8 h blue light exposure. Blue light-induced retinal degeneration was suppressed by *trp* mutations that prevent phototransduction-associated calcium influx, and by reducing oxidative stress. One-day-old flies did not exhibit blue light-dependent oxidative stress or retinal degeneration. **b** Schematic for photoreceptor transcriptome profiling after exposure to blue light. Male *cn*, *bw; Rh1*-*Gal4*, *UAS*-*GFP*-*Msp300KASH* flies were raised in 12 h/12 h light/dark conditions for 1 or 6 days prior to 3 h blue light exposure (2 mW/cm^2^) or dark control. A custom designed optical stimulator with built-in temperature control was used for all experiments. Photoreceptor nuclei labeled with KASH-GFP were affinity isolated and nuclear RNA was ribo-depleted and analyzed by RNA-seq

Although blue light induces retinal degeneration in mature flies, our previous results showed that very young flies are resilient to longer periods of blue light (Fig. 1a). Newly-eclosed flies, that have recently emerged from the pupal case and are less than one day old, did not undergo retinal degeneration in response to prolonged blue light [19]. In contrast, mature flies that are only six days old, underwent severe retinal degeneration when exposed to the same level of blue light [19]. Blue light-induced retinal degeneration required an intact phototransduction pathway and calcium influx, mediated by the transient receptor potential (*trp*) calcium channel [19]. Since blue light provides a model for light-induced lipid peroxidation in the eye, we sought to identify the gene regulatory mechanisms utilized by *Drosophila* photoreceptors to cope with the oxidative stress resulting from blue light exposure. Here, we profiled the transcriptome of *Drosophila* photoreceptors following short blue light exposure at different ages to gain insight into neuroprotective pathways that enable photoreceptors to withstand light-induced oxidative stress.

## Results

### Blue light induces neuroprotective gene expression changes in photoreceptors

To identify gene regulatory mechanisms involved in the response of photoreceptors to blue light-induced oxidative stress, we profiled the transcriptome of photoreceptor cells in flies that were exposed to blue light relative to dark control. Here, we exposed flies to 3 h blue light, which we previously showed was sufficient to increase levels of reactive oxygen species in the eye of six-day-old flies, but not in one-day-old flies [19]. This shorter 3 h blue light exposure resulted in less than 1% rhabdomere loss at both ages (Additional file 1: Figure S1), enabling us to isolate intact photoreceptor nuclei for RNA-seq analysis. To isolate photoreceptor nuclear RNA, we used previously developed methods to affinity-purify *Rh1*-*Gal4 *>* KASH*-*GFP* tagged nuclei from R1–R6 cells in adult heads [20, 21]. Since white-eyed flies are sensitized to blue light [9], we depleted eye pigments from *Rh1*-*Gal4 *>* KASH*-*GFP* flies, which have red eyes due to the presence of the *mini*-*white* transgene marker, by introducing homozygous mutations for *cn* and *bw* [22, 23]. We then exposed one- or six-day-old flies to 3 h of blue light and isolated photoreceptor nuclear RNA for RNA-seq analysis (Fig. 1b).

To test the enrichment of photoreceptor transcripts using our affinity-isolation procedure, we compared the transcriptome of the whole head homogenate (pre-isolation) and post-isolation sample from the control dark treated day one flies. Consistent with previous results using this affinity-isolation approach [20], the post-isolation samples differed substantially from the pre-isolation samples based on the principal component analysis (Additional file 1: Figure S2A). We identified 521 genes, including GFP, as significantly enriched using edgeR analysis (False Discovery Rate, FDR < 0.05, Fold change, FC > 2) in the post-isolation samples relative to the pre-isolation samples (Additional file 1: Figure S2B, Additional file 2: Table S1). These genes were enriched for Gene Ontology (GO) terms associated with photoreceptor development and function (Additional file 3: Table S2). Thus, we conclude that our post-isolation samples are enriched for photoreceptor-expressed transcripts.

**Fig. 2:**
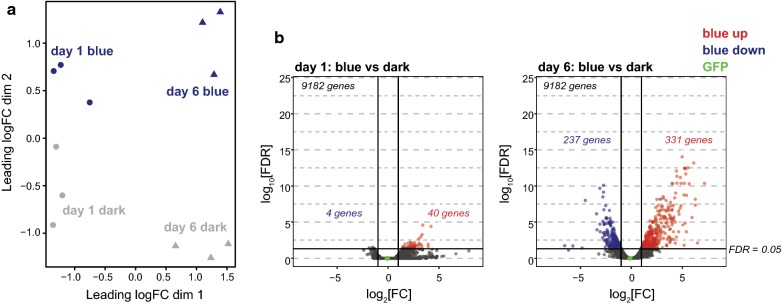
Blue light exposure alters expression of stress response, photoreceptor development, and circadian rhythm genes in six-day-old photoreceptors. **a** Multidimensional scaling plot of distances between gene expression profiles based on log_2_ fold change. The plot shows three biological replicates for affinity-enriched photoreceptor nuclear RNA from male day one or day six flies exposed to 3 h blue light or 3 h dark (control). **b** Volcano plots showing the differential gene expression profiles in day one (left panel) or day six (right panel) photoreceptors induced by blue light relative to dark (control). Fold change was plotted as log_2_(fold change) for each gene relative to its false discovery rate (−log_2_[FDR]). Genes with significantly differential expression (FDR < 0.05) are highlighted in red or blue, and GFP is shown in green for comparison

Next, we compared the photoreceptor-enriched transcriptome of day one and day six flies that had been exposed to blue light or the dark control. Multidimensional scaling plots revealed that both age and light treatment influenced the variation in gene expression between the samples, with the three biological replicates for each treatment and age grouping together (Fig. 2a). To identify genes that showed altered expression profiles upon blue light treatment, we used edgeR analysis to identify differentially expressed genes in blue versus dark treated samples from day one or day six flies. Only 40 and four genes were significantly up- or downregulated (FDR < 0.05), respectively, in day one photoreceptors upon blue light stress (Fig. 2b). In contrast, 331 and 237 genes were significantly up- or downregulated, respectively, in day six photoreceptors upon blue light stress (Fig. 2b). Only six genes were uniquely regulated in response to blue light in day one photoreceptors, and most of these genes also showed strong, albeit not significant, fold changes in gene expression in day six flies (Additional file 1: Fig. S3). These data indicate that six-day-old flies exhibit substantial gene expression changes in photoreceptors in response to blue light, whereas these gene expression changes are largely absent in newly-eclosed flies. We previously observed that in contrast to six-day-old flies, one-day-old flies did not show increased levels of reactive oxygen species upon blue light exposure [19]. Together, these observations suggest that one-day-old flies experience much lower levels of blue light-induced oxidative stress than mature, six-day-old flies.

Next, we asked if the gene expression changes that we observed in response to blue light in day six flies could be neuroprotective since 3 h blue light exposure increased oxidative stress levels in the eye but did not cause retinal degeneration (Additional file 1: Fig. S1). GO term enrichment analysis revealed that pathways associated with the response to unfolded proteins, environmental stresses such as heat, ion transport and protein translation were upregulated in response to blue light exposure in six-day-old flies (Table 1, Additional file 3: Table S2). The blue light-upregulated genes included many heat shock protein genes such as *Hsc70*-*2*, *Hsc70*-*3*, *Hsc70*-*5*, *Hsp68*, *Hsp70Aa* and *Hsp70Bc* that are part of the Heat Shock Protein 70 superfamily of chaperones. These chaperones are upregulated in response to chemical and thermal stress, resolve misfolded and aggregated proteins, and are implicated in having a protective role in neurodegenerative disease [24]. In addition, several genes encoding proteins involved in ion transport were upregulated in response to blue light. These genes include mitochondrial transporters such as *Thiamine pyrophosphate carrier protein 1* (*Tpc1*) and *CG5646*, several putative organic cation transporters such as *CG14855*, *CG14856* and *SLC22A*, and the gap junction protein *Innexin 7 (Inx7*), which together might restore calcium and energy homeostasis within photoreceptors following blue light exposure. Several genes associated with protein translation were also upregulated in response to blue light including several cytoplasmic aminoacyl-tRNA synthetases (e.g. *GluProRS/Aats*-*glupro*, *GlyRS/Aats*-*gly*, *TrpRS/Aats*-*trp*). Specialized translation is associated with the stress response [25], but increased translation following blue light might also be required to restore Rh1 levels, which are depleted due to endocytosis of activated metarhodopsin [14, 16]. Although DNA repair was not identified in the GO term enrichment analysis, several genes associated with repair of DNA damage were upregulated in response to blue light including *DNA ligase III* (*lig3*), *mutagen*-*sensitive 205* (*mus205*), *Replication Protein A 70* (*RpA*-*70*), *Inverted repeat*-*binding protein* (*Irbp*), *Inverted repeat binding protein 18* *kDa* (*Irbp18*), *Replication factor C subunit 4* (*RfC4*), *Xrp1*, *nbs*, and *CG3448*. Thus, blue light exposure initiates a transcriptional stress response in photoreceptors that induces repair mechanisms to combat protein misfolding and DNA damage, and to restore Rh1 levels and ion homeostasis.

**Table 1:** Enriched biological process GO terms identified for day 6 blue versus dark upregulated genes

GO term	Description	p value	FDR	Enrichment	Genes
GO:0006418	tRNA aminoacylation for protein translation	4.50E − 06	0.00646	6.82	*Aats*-*glupro*, *CG10802*, *Aats*-*thr*, *Aats*-*gly*, *Aats*-*cys*, *CG33123*, *Aats*-*trp*, *CG17259*, *Aats*-*asp*
GO:0006399	tRNA metabolic process	0.000895	0.292	3.06	*Aats*-*glupro*, *CG10802*, *CG6353*, *Aats*-*thr*, *Aats*-*gly*, *CG33123*, *Aats*-*cys*, *Aats*-*trp*, *CG17259*, *CG18596*, *Aats*-*asp*
GO:0006820	Anion transport	0.000111	0.0532	3.05	*CG14857*, *CG14856*, *CG5535*, *CG7589*, *CG14855*, *CG5802*, *CG13646*, *CG5646*, *JhI*-*21*, *CG9864*, *CG42575*, *w*, *MFS3*, *Tpc1*, *CG7442*
GO:0015695	Organic cation transport	0.000128	0.0574	13.47	*CG5646*, *CG3476*, *CG7442*, *Tpc1*
GO:0015696	Ammonium transport	0.000465	0.167	10.1	*CG5646*, *w*, *CG3476*, *CG7442*
GO:0009631	Cold acclimation	0.000338	0.143	18.18	*Hsp23*, *Hsp26*, *Hsp83*
GO:0006457	Protein folding	0.00042	0.159	3.13	*Hsp68*, *Hsp23*, *CG14894*, *Hsp70Bc*, *Hsp26*, *Hsc70*-*3*, *Hsc70*-*5*, *Hsp70Aa*, *Hsc70*-*2*, *Hsp83*, *wbl*, *CG5525*
GO:0042026	Protein refolding	2.37E−08	0.00017	14.26	*Hsp68*, *Hsp23*, *Hsp26*, *Hsp70Bc*, *Hsc70*-*3*, *Hsc70*-*5*, *Hsc70*-*2*, *Hsp70Aa*
GO:0061077	Chaperone-mediated protein folding	8.51E−06	0.00555	6.34	*Hsp68*, *Hsp23*, *Hsp26*, *Hsp70Bc*, *Hsc70*-*3*, *Hsc70*-*5*, *Hsc70*-*2*, *Hsp70Aa*, *CG5525*
GO:0009408	Response to heat	0.000101	0.0516	4.27	*Hsp68*, *Hsp23*, *Nup98*-*96*, *Hsp26*, *Hsp70Bc*, *Hsc70*-*3*, *Hsc70*-*5*, *Hsc70*-*2*, *Hsp70Aa*, *Hsp83*
GO:0006986	Response to unfolded protein	7.39E−06	0.00589	11.36	*Hsp68*, *Hsp70Bc*, *Hsc70*-*3*, *Hsc70*-*5*, *Hsc70*-*2*, *Hsp70Aa*
GO:0006458	‘de novo’ protein folding	1.13E−05	0.00626	8.48	*Hsp68*, *Hsp70Bc*, *Hsc70*-*3*, *Hsc70*-*5*, *Hsc70*-*2*, *Hsp70Aa*, *CG5525*
GO:0051085	Chaperone cofactor-dependent protein refolding	2.93E−06	0.00525	12.99	*Hsp68*, *Hsp70Bc*, *Hsc70*-*3*, *Hsp70Aa*, *Hsc70*-*2*, *Hsc70*-*5*
GO:0034605	Cellular response to heat	8.56E−06	0.00511	7.35	*Hsp68*, *Nup98*-*96*, *Hsp70Bc*, *Hsc70*-*3*, *Hsc70*-*5*, *Hsc70*-*2*, *Hsp70Aa*, *Hsp83*
GO:0035080	Heat shock-mediated polytene chromosome puffing	0.000338	0.135	18.18	*Nup98*-*96*, *Hsp70Bc*, *Hsp70Aa*

In addition to the genes that were upregulated in response to blue light, a similar number of genes were downregulated in response to blue light exposure in day six, but not day one, flies. Intriguingly, these blue light-downregulated genes were enriched for GO terms related to photoreceptor function and phototransduction including regulation of membrane potential, rhodopsin metabolism, and response to light stimulus (Table 2, Additional file 3: Table S2). Several genes involved in regulating membrane potential were downregulated in response to blue light including potassium and chloride channels and their regulators such as *Chloride channel*-*a* (*ClC*-*a*), *Slowpoke* (*slo*), *Shaker* (*Sh*), *small conductance calcium*-*activated potassium channel* (*SK*), *ether a go*–*go* (*eag*), *Slip1*, *Na*^+^-*driven anion exchanger 1* (*Ndae1*) and *Hyperkinetic* (*Hk*). In addition, factors involved in post-translational modification and maturation of rhodopsin such as *Hexosaminidase 1* (*Hexo1*), *alpha*-*Mannosidase class II b* (*alpha*-*Man*-*IIb*), and *fused lobes* (*fdl*) were downregulated in response to blue light. Most strikingly, several genes with well-characterized roles in phototransduction were significantly downregulated in day six flies upon blue light exposure. These genes include components of the phototransduction machinery such as *retinal degeneration A* (*rdgA*), *retinal degeneration C* (*rdgC*), *Histidine decarboxylase* (*Hdc*), *Calcium*, *integrin binding family member 2* (*Cib2*), and the calcium channel *trp*. Several other genes involved in voltage-gated calcium influx into photoreceptors were also downregulated in response to blue light including *Ca*^*2*+^-*channel protein alpha*^*1*^ *subunit D* (*Ca*-*alpha1D*), *Ca*^*2*+^-*channel*-*protein*-*beta*-*subunit* (*Ca*-*beta*), and *olf186*-*F*, which encodes a subunit of the store-operated calcium entry channel. Previously, we showed that blue light-induced retinal degeneration required an intact phototransduction pathway and Trp-mediated calcium influx [19]. Here, our data suggest that under phototoxic conditions, photoreceptors downregulate expression of phototransduction components and calcium channels, potentially as part of a neuroprotective response to mitigate the calcium influx resulting from light exposure.

**Table 2:** Enriched biological process GO terms identified for day 6 blue versus dark downregulated genes

GO term	Description	p value	FDR	Enrichment	Genes
GO:0009886	Post-embryonic animal morphogenesis	0.000318	0.127	2.49	*app*, *ewg*, *mirr*, *ara*, *oc*, *so*, *dlg1*, *sd*, *Cbl*, *jumu*, *CG30456*, *psq*, *RhoGEF2*, *Exn*, *mthl1*, *CG33275*, *zfh2*, *CG13366*
GO:0009653	Anatomical structure morphogenesis	0.00041	0.134	1.77	*app*, *kek4*, *ewg*, *oc*, *vri*, *dlg1*, *dnt*, *ric8a*, *Cbl*, *jumu*, *csw*, *RhoGEF2*, *Prosap*, *mthl1*, *Moe*, *CG13366*, *zfh2*, *Hr39*, *slik*, *CHES*-*1*-*like*, *Shroom*, *fru*, *mirr*, *CG13188*, *caup*, *ara*, *so*, *gl*, *sd*, *psq*, *CG30456*, *Crg*-*1*, *fred*, *pyd*, *Exn*, *CG33275*
GO:0042693	Muscle cell fate commitment	0.000539	0.133	42.96	*caup*, *ara*
GO:0006357	Regulation of transcription by RNA polymerase II	0.000989	0.177	1.97	*CHES*-*1*-*like*, *mirr*, *ewg*, *Mef2*, *fru*, *caup*, *ara*, *oc*, *dlg1*, *gl*, *so*, *sd*, *onecut*, *psq*, *Eip74EF*, *Crg*-*1*, *NfI*, *csw*, *jing*, *tim*, *jigr1*, *Camta*, *Hr39*, *Elp3*
GO:0006355	Regulation of transcription, DNA-templated	3.00E−04	0.154	1.8	*CTCF*, *ewg*, *Kdm4B*, *tinc*, *oc*, *vri*, *dlg1*, *jumu*, *onecut*, *Eip74EF*, *csw*, *NfI*, *tim*, *zfh2*, *Hr39*, *Elp3*, *Pdp1*, *CHES*-*1*-*like*, *fru*, *Mef2*, *mirr*, *CG13188*, *caup*, *ara*, *Hmt4*-*20*, *Hmx*, *gl*, *so*, *sd*, *psq*, *Crg*-*1*, *jing*, *jigr1*, *Camta*, *wts*, *thoc5*
GO:0030001	Metal ion transport	0.000378	0.135	3.67	*eag*, *Hk*, *Ca*-*alpha1D*, *Ndae1*, *Ca*-*beta*, *Sh*, *SK*, *trp*, *olf186*-*F*, *slo*
GO:0042391	Regulation of membrane potential	2.52E−05	0.0903	5.05	*eag*, *inaF*-*D*, *Ca*-*alpha1D*, *Prosap*, *Sh*, *inaF*-*C*, *SK*, *Slob*, *Moe*, *slo*
GO:0007619	Courtship behavior	0.000837	0.162	9.04	*eag*, *rut*, *Sh*, *gb*
GO:0048150	Behavioral response to ether	0.000539	0.138	42.96	*eag*, *Sh*
GO:0007617	Mating behavior	5.54E−05	0.0993	4.62	*eag*, *tim*, *rut*, *fru*, *Sh*, *gb*, *dlg1*, *Moe*, *Hr39*, *slo*
GO:0007275	Multicellular organism development	0.000177	0.141	3.25	*ewg*, *fru*, *Mef2*, *mirr*, *CG2681*, *oc*, *vri*, *dlg1*, *dnt*, *cdi*, *Elp3*, *Pdp1*, *Sema*-*1b*
GO:0046154	Rhodopsin metabolic process	4.33E−05	0.104	11.93	*fdl*, *rdgA*, *alpha*-*Man*-*IIb*, *trp*, *Hexo1*
GO:0001745	Compound eye morphogenesis	0.000177	0.127	4.44	*fred*, *mirr*, *caup*, *ara*, *pyd*, *oc*, *so*, *gl*, *sd*
GO:0008049	Male courtship behavior	0.000892	0.168	5.26	*fru*, *gb*, *dlg1*, *Moe*, *Hr39*, *slo*
GO:0045433	Male courtship behavior, veined wing generated song production	0.000837	0.167	9.04	*fru*, *Moe*, *Hr39*, *slo*
GO:0045938	Positive regulation of circadian sleep/wake cycle, sleep	0.000122	0.124	14.32	*Hk*, *homer*, *Sh*, *mld*
GO:0045187	Regulation of circadian sleep/wake cycle, sleep	0.000344	0.13	7.95	*Hk*, *tim*, *homer*, *mld*, *Sh*
GO:0042752	Regulation of circadian rhythm	0.000248	0.148	4.77	*Hk*, *tim*, *homer*, *mld*, *Sh*, *CG33275*, *gl*, *so*
GO:0007623	Circadian rhythm	0.000404	0.138	3.99	*Hk*, *tim*, *Mef2*, *dlg1*, *vri*, *so*, *gl*, *Pdp1*, *slo*
GO:0016057	Regulation of membrane potential in photoreceptor cell	0.000638	0.147	16.11	*inaF*-*D*, *SK*, *Moe*
GO:1902680	Positive regulation of RNA biosynthetic process	0.000803	0.175	2.37	*Mef2*, *mirr*, *caup*, *ara*, *oc*, *gl*, *so*, *sd*, *jumu*, *onecut*, *Eip74EF*, *NfI*, *jing*, *Camta*, *thoc5*, *Hr39*, *Pdp1*
GO:0035120	Post-embryonic appendage morphogenesis	0.000543	0.13	3.26	*mirr*, *ara*, *Exn*, *mthl1*, *CG33275*, *sd*, *zfh2*,, *Cbl*, *jumu*, *CG30456*, *psq*
GO:0045317	Equator specification	0.000236	0.154	21.48	*mirr*, *caup*, *ara*
GO:0009887	Animal organ morphogenesis	0.000159	0.143	2.72	*mirr*, *ewg*, *CG13188*, *caup*, *ara*, *oc*, *gl*, *so*, *vri*, *sd*, *dnt*, *fred*, *pyd*, *Prosap*, *mthl1*, *CG13366*, *Hr39*
GO:0045935	Positive regulation of nucleobase-containing compound metabolic process	0.00072	0.161	2.32	*mirr*, *Mef2*, *caup*, *ara*, *oc*, *gl*, *so*, *sd*, *jumu*, *tankyrase*, *onecut*, *Eip74EF*, *NfI*, *jing*, *Camta*, *thoc5*, *Hr39*, *Pdp1*
GO:0007635	Chemosensory behavior	8.68E−05	0.124	4.38	*mura*, *smi35A*, *gish*, *rut*, *Sh*, *gb*, *nord*, *Moe*, *trp*, *psq*
GO:0007610	Behavior	2.40E−05	0.172	2.36	*nord*, *oc*, *dlg1*, *vri*, *hppy*, *CG13192*, *eag*, *smi35A*, *gish*, *tim*, *Sh*, *Prosap*, *mld*, *Moe*, *Elp3*, *Hr39*, *Hk*, *Mef2*, *fru*, *gb*, *trp*, *psq*, *slo*, *mura*, *t*, *homer*, *rut*
GO:0035025	Positive regulation of Rho protein signal transduction	0.000317	0.134	11.45	*RhoGEF2*, *Exn*, *CG33275*, *CG30456*
GO:0009314	Response to radiation	5.00E−04	0.138	3.09	*smi35A*, *tim*, *CG30118*, *rdgA*, *CG9236*, *Sh*, *Camta*, *dlg1*, *wts*, *gl*, *Hdc*, *trp*
GO:0009416	Response to light stimulus	0.000275	0.152	3.53	*smi35A*, *tim*, *rdgA*, *CG30118*, *CG9236*, *Sh*, *Camta*, *dlg1*, *gl*, *Hdc*, *trp*

### Blue light-induced changes in gene expression show different temporal profiles

**Fig. 3:**
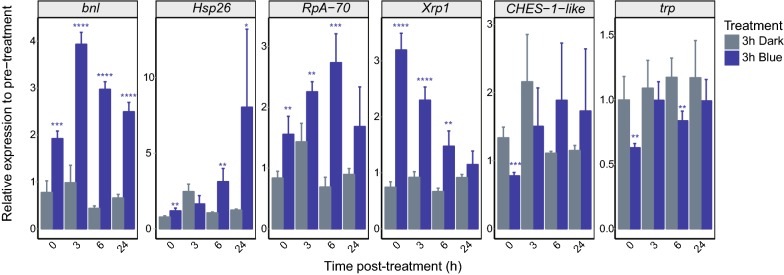
Blue light-induced changes in gene expression are transient. Six-day-old male *cn*, *bw*; *Rh1*-*Gal4*, *UAS*-*GFP*-*Msp300KASH* flies were exposed to 3 h blue light exposure or dark control, and gene expression was analyzed in dissected eyes at 0, 3, 6 or 24 h following treatment by qPCR. Expression is shown relative to the geometric mean of *RpL32* and *eIF1A* and is normalized to the pre-treatment sample, which is set to one. *p* values, *t* test between blue treatment and dark control at the same time post-treatment (*p < 0.05; **p < 0.01, ***p < 0.001, ****p < 0.0001; n = 4)

Exposure to moderate levels of stress protects photoreceptors against retinal degeneration [26]. To test if exposure to light stress would increase basal expression levels of stress response genes, we asked if the changes in gene expression that occurred in photoreceptors in response to blue light returned to pre-treatment levels after different intervals of dark exposure, post light-treatment. To do this, we exposed male six-day-old *cn bw; Rh1*-*Gal4 *>* KASH*-*GFP* flies to 3 h blue light or dark control, and then incubated flies for 0, 3, 6 or 24 h in the dark. We then dissected eyes and examined expression of several blue light-regulated genes using qPCR. We normalized expression of each gene to the pre-treatment control, and compared relative expression levels between the blue and dark samples for each time point. We examined four blue light-induced genes, *branchless* (*bnl*), *Heat shock protein 26* (*Hsp26)*, *RpA*-*70* and *Xrp1* and two blue light-repressed genes, *Checkpoint suppressor 1*-*like* (*CHES*-*1*-*like)* and *trp* (Fig. 3). The four upregulated genes all showed different expression profiles following exposure to 3 h blue light: *Xrp1* and *RpA*-*70* showed significantly increased expression in blue light versus dark control at 0, 3 and 6 h post-treatment, but returned to basal levels by 24 h post-treatment. In contrast, *bnl* and *Hsp26* levels remained high 24 h after blue light exposure. The two downregulated genes, *CHES*-*1*-*like* and *trp*, showed significantly decreased expression levels immediately post-treatment (0 h) but returned to basal levels by 3 h post-treatment. These data indicate that blue light-repression of genes is transient and might require continual exposure to the light source. In contrast, exposure to blue light increases expression of stress response genes, some of which remain at relatively high levels up to 1 day after flies are removed from the source of light stress.

### An intact phototransduction pathway and calcium influx are required for blue light-induced upregulation of stress response genes, but not downregulation of visual function genes

**Fig. 4:**
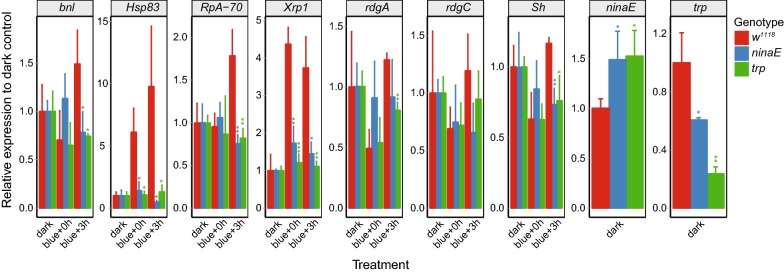
An intact phototransduction pathway and calcium influx are required for blue light-induced upregulation of stress response genes, but not downregulation of visual function genes. Six-day-old male *w*^*1118*^, *ninaE*^*7*^ and *trp*^*9*^ flies were exposed to 3 h blue light or dark control, and gene expression was analyzed in dissected eyes at 0 or 3 h following treatment by qPCR. Expression is shown relative to the geometric mean of *RpL32* and *eIF1A* and is normalized to the dark control for each genotype, which is set to one. *p* values, *t* test between *ninaE*^*7*^ or *trp*^*9*^ and *w*^*1118*^ at the same time post-treatment (*p < 0.05; **p < 0.01, ***p < 0.001; n = 3)

Phototransduction in R1–R6 photoreceptors initiates with the light-sensing G-protein coupled receptor, Rhodopsin 1 (Rh1 encoded by *ninaE*), and culminates in calcium influx, largely mediated by the Trp channel [11]. We previously showed that blue light-induced retinal degeneration requires both phototransduction and calcium influx because rhabdomere loss was suppressed by mutations that reduce Rh1 protein levels to ~ 1% of wild-type levels (*ninaE*^*7*^) [27] or reduce Trp expression (*trp*^*9*^) [19]. To test if phototransduction and calcium influx were necessary for blue light-regulated gene expression changes, we examined expression of blue light-regulated genes in eyes from *ninaE*^*7*^ or *trp*^*9*^ flies. We compared gene expression to white-eyed *w*^*1118*^ flies, which lack eye pigment but have otherwise normal phototransduction. We exposed six-day-old male flies of each genotype to 3 h blue light and examined gene expression relative to the dark control at either 0 or 3 h post-treatment by qPCR in dissected eyes (Fig. 4). We examined four blue light-upregulated genes, *bnl*, *Heat shock protein 83* (*Hsp83*), *RpA*-*70* and *Xrp1*, and three downregulated genes, *retinal degeneration A* (*rdgA*), *retinal degeneration C* (*rdgC*) and *Shaker* (*Sh*). Blue light exposure resulted in increased expression of *bnl*, *Hsp83*, *RpA*-*70* and *Xrp1* either at 0 or 3 h post-treatment in *w*^*1118*^ flies, and mutations in *ninaE* and *trp* suppressed this increase (Fig. 4). In contrast, *ninaE and trp* mutations did not suppress the downregulation of *rdgA*, *rdgC* or *Sh* upon blue light exposure. We did not observe significant differences in basal levels of expression of any of the seven blue-light regulated genes tested between *w*^*1118*^, *ninaE* and *trp* flies in the dark controls relative to the pre-treatment samples (data not shown). We note that while *trp* expression was significantly reduced in *ninaE* flies, calcium influx is already suppressed in *ninaE* mutants because Rh1 functions upstream of the Trp channel in the phototransduction cascade. Together, these data indicate that the blue light-induced and repressed genes are regulated via distinct pathways. Blue light-upregulated genes require an intact phototransduction cascade and calcium influx, whereas blue light-repressed genes do not. Instead, blue light-downregulated genes are repressed only immediately after light exposure, suggesting that light itself might be involved in the transient repression of these genes.

### Developmental transitions in photoreceptor gene expression correlate with the differential susceptibility to blue light between day one and six

**Fig. 5:**
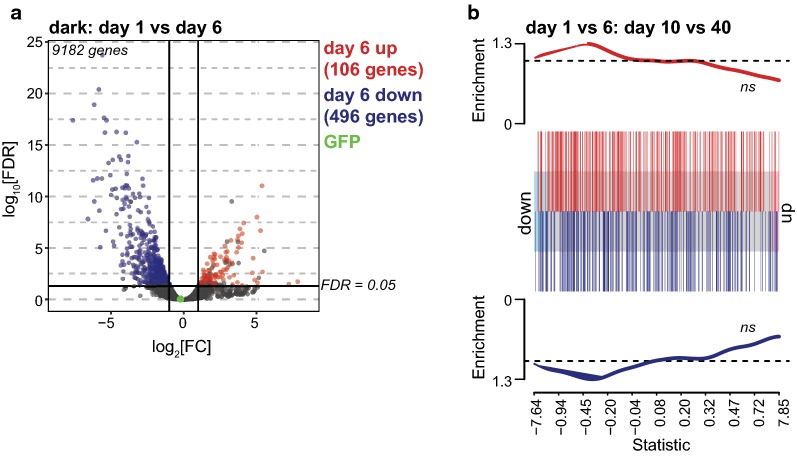
Gene expression changes in photoreceptors between day one and six represent developmental transitions. **a** Volcano plot showing the differential gene expression profiles in the control (dark-treated) day one versus day six photoreceptors. Fold change was plotted as log_2_(fold change) for each gene relative to its false discovery rate (−log_2_[FDR]). Genes with significantly differential expression (FDR < 0.05) are highlighted in red or blue, and GFP is shown in green for comparison. **b** Gene set analysis barcode plot overlaying RNA-seq data from day one versus day six photoreceptors with age-regulated genes in photoreceptors between day 10 and 40. Day one versus day six data are shown as a shaded rectangle with genes horizontally ranked by moderated *t*-statistic, upregulated genes shaded in pink, and downregulated genes shaded in blue. Previously described age-regulated genes are overlaid as red (age-upregulated) or blue (age-downregulated) bars. Red and blue traces above and below the barcode represent relative enrichment. FDR values represent overlap in the same direction using the roast method; *ns* not significant

Since we did not observe substantial changes in gene expression upon blue light exposure in day one flies, we next wondered if underlying changes in gene expression between day one and day six photoreceptors could account for the differential susceptibility to blue light. Supporting this hypothesis, day one flies have lower basal levels of hydrogen peroxide than day six flies, even prior to blue light exposures [19]. Principal component analysis of the blue and dark treated RNA-seq samples revealed that both light treatment and age contributed to differences in the gene expression profile (Fig. 2a). Indeed, we identified 106 and 496 genes that were significantly up- or downregulated, respectively, between day one and day six in photoreceptors in the absence of blue light exposure (Fig. 5a). Importantly, we did not observe differences in GFP expression between day one and day six samples (Fig. 5a). Further, we did not observe any differences in enrichment of GFP in day one versus day six affinity purifications based on qPCR (data not shown). Thus, affinity-enrichment of photoreceptor nuclear RNA was not affected by differences in age.

Next, we asked if the changes in gene expression between day one and day six resembled those gene expression changes observed in aging photoreceptors. We compared the gene expression changes observed between day one and day six in *cn bw; Rh1*-*Gal4 *>* KASH*-*GFP* flies with those observed between day 10 and 40 in pigmented male *Rh1*-*Gal4 *>* KASH*-*GFP* flies [20]. To do this, we performed gene set enrichment analysis to compare the gene expression changes between day one and six with day 10 and 40, and asked if these expression changes showed significant enrichment in either direction. We did not observe any significant enrichment of either up- or downregulated genes between day one and six, and day 10 and 40 (Fig. 5b). Thus, the gene expression changes that occur between day one and six in photoreceptors differ from those observed during later stages of the aging process in photoreceptors, suggesting that these gene expression changes between day one and six do not reflect aging. Consistent with these observations, white-eyed flies show peak reproductive capacity between 3 and 6 days post-eclosion [28]. Moreover, the fly strains used in our experiments show maximum life spans of up to 80 days under our growth conditions at 25 °C [20]. Together, these data suggest that the changes in gene expression between early post-eclosion at day one and day six do not represent aging.

Instead, we wondered if the changes in gene expression between day one and day six represented developmental transitions between newly-eclosed flies and mature, young adults. Strikingly, almost five times as many genes were downregulated between day one and day six as compared with upregulated genes. Whereas the genes that are upregulated between day one and day six were enriched for several stress-related pathways including response to hypoxia, defense response, and heat response (Table 3), the downregulated genes were enriched for pathways associated with photoreceptor and/or eye development (Table 4). We observed reduced expression of genes involved in Notch signaling such as *Notch* (*N*), *Delta* (*Dl*), *Serrate* (*Ser*) and *fringe* (*fng*). Notch signaling plays an important role during eye development and specification of photoreceptor fate [29, 30], and our data suggest that newly-eclosed flies still show some activity of this pathway, but that this rapidly declines over the first few days post-eclosion. We next asked if some of these changes in gene expression could reduce the ability of day six flies to withstand blue light exposure. Indeed, some of the genes that were downregulated in the first week of life could account for the increased susceptibility of older flies to blue light. For example, day six flies showed reduced expression of *Calphotin* (*Cpn*), encoding an immobile calcium buffer required for rhabdomere development [31]. *Cpn* hypomorph files develop light-induced retinal degeneration [13], suggesting that reductions in *Cpn* expression could reduce the ability of six-day-old flies to buffer the increased calcium levels that are necessary for blue light-induced retinal degeneration [19]. In addition, day six flies showed reduced expression of several genes with important roles in maintaining cellular redox homeostasis including *Peroxidase* (*Pxd*), which converts hydrogen peroxide to water. Moreover, 10 of the 96 annotated Cytochrome P450 genes (*Cyp28d1*, *Cyp317a1*, *Cyp4c3*, *Cyp4e1*, *Cyp4e3*, *Cyp4s3*, *Cyp6a20*, *Cyp6a8*, *Cyp6a9*, and *Cyp9b1*) were downregulated between day one and day six. The upregulation of stress-related pathways between day one and six suggests that photoreceptors experience considerable stress as a normal part of their early life, potentially resulting from exposure to white light. In addition, the downregulation of many genes involved in signaling and developmental processes supports the idea that major developmental transitions occur in photoreceptors between the late pupal/newly-eclosed adult and mature-young adult stage. We propose that these collective changes in gene expression in the first week of adult life diminish the capacity of photoreceptors to maintain homeostasis under phototoxic conditions, resulting in their susceptibility to blue light-induced retinal degeneration.

**Table 3:** Enriched biological process GO terms identified for day 6 versus day 1 upregulated genes

GO term	Description	p value	FDR	Enrichment	Genes
GO:0055093	Response to hyperoxia	0.000213	0.0899	24.23	*AttA*, *AttB*, *DptB*
GO:0050830	Defense response to Gram-positive bacterium	6.04E−05	0.0394	11.69	*AttA*, *Dro*, *AttB*, *TotM*, *DptB*
GO:0009617	Response to bacterium	1.38E−05	0.0142	5.42	*AttA*, *Dro*, *Lectin*-*galC1*, *cathD*, *TotM*, *AttB*, *DptB*, *TotX*, *TotA*, *TotC*
GO:0051704	Multi-organism process	5.45E−07	0.000977	4.36	*AttA*, *Drsl4*, *Dro*, *cathD*, *TotM*, *AttB*, *TotX*, *TotC*, *jumu*, *Est*-*6*, *Npl4*, *Lectin*-*galC1*, *CG34215*, *DptB*, *Drsl5*, *TotA*
GO:0051707	Response to other organism	1.02E−07	0.000732	5.31	*AttA*, *Drsl4*, *Dro*, *cathD*, *TotM*, *AttB*, *TotX*, *TotC*, *jumu*, *Npl4*, *Lectin*-*galC1*, *CG34215*, *DptB*, *Drsl5*, *TotA*
GO:0019731	Antibacterial humoral response	2.85E−06	0.00408	21.15	*AttA*, *Lectin*-*galC1*, *Dro*, *AttB*, *DptB*
GO:0098542	Defense response to other organism	1.05E−05	0.0125	5.01	*AttA*, *Lectin*-*galC1*, *Dro*, *Drsl4*, *cathD*, *AttB*, *TotM*, *CG34215*, *Drsl5*, *DptB*, *jumu*
GO:0030431	Sleep	4.99E−05	0.0397	6.02	*bgm*, *AttA*, *Cyp6g1*, *CG8435*, *CG8329*, *Iris*, *Amy*-*p*, *CG16926*
GO:0006952	Defense response	5.33E−05	0.0382	3.88	*CG10433*, *AttA*, *Lectin*-*galC1*, *Dro*, *Drsl4*, *cathD*, *AttB*, *TotM*, *CG34215*, *Drsl5*, *DptB*, *jumu*
GO:0009605	Response to external stimulus	2.34E−05	0.021	2.97	*CG6188*, *AttA*, *Drsl4*, *Dro*, *cathD*, *AttB*, *TotM*, *TotX*, *Slob*, *TotC*, *jumu*, *Npl4*, *Lectin*-*galC1*, *CG9236*, *CG34215*, *DptB*, *Drsl5*, *TotA*
GO:1901607	Alpha-amino acid biosynthetic process	0.000826	0.296	9.35	*CG6188*, *CG5840*, *CG10184*, *CG1315*
GO:0009109	Coenzyme catabolic process	0.000125	0.0749	88.84	*CG6188*, *CG8665*
GO:0006805	Xenobiotic metabolic process	0.000156	0.0747	26.65	*Cyp6g1*, *St1*, *CG17322*
GO:0046689	Response to mercury ion	0.000125	0.0691	88.84	*Cyp6g1*, *TotA*
GO:0034605	Cellular response to heat	0.000478	0.19	10.77	*TotM*, *TotX*, *TotA*, *TotC*

**Table 4:** Enriched biological process GO terms identified for day 6 versus day 1 downregulated genes

GO term	Description	p value	FDR	Enrichment	Genes
GO:0032502	Developmental process	6.95E−12	6.23E−09	1.65	*5*-*HT2*, *Inx2*, *CG9634*, *e*, *CG17211*, *Mdr65*, *sas*, *fz*, *Mmp1*, *dlp*, *Cht5*, *uif*, *cv*-*2*, *rpr*, *Acp65Aa*, *W*, *aret*, *fng*, *N*, *Sb*, *pk*, *spz5*, *Cry*, *vkg*, *l(3)mbn*, *Phk*-*3*, *scb*, *Aph*-*4*, *Cpr66D*, *knk*, *RhoGAP15B*, *Cpr100A*, *Cpr49Ac*, *Pkg21D*, *ETHR*, *Cpr49Ah*, *Cpr49Af*, *ec*, *Cpr49Ae*, *how*,, *ds*, *Sema*-*5c*, *LanB1*, *Fas2*, *dnd*, *grh*, *stl*, *out*, *TwdlT*, *Cad74A*, *esg*, *Cpr47Ea*, *miple2*, *blot*, *melt*, *DAAM*, *Cg25C*, *fj*, *Ccp84Ad*, *drd*, *Ccp84Ab*, *bnb*, *CG31475*, *spz3*, *TwdlE*, *Ccp84Aa*, *Cpr62Bc*, *Cpr62Bb*, *kkv*, *Cpr73D*, *Dl*, *qsm*, *aay*, *prc*, *Cht2*, *pio*, *ple*, *d*, *dp*, *CG10348*, *fw*, *Pxd*, *pot*, *Duox*, *wdp*, *Gp150*, *serp*, *verm*, *pbl*, *Ser*, *Gasp*, *Sobp*, *Tie*, *mys*, *scaf*, *laccase2*, *Cpr97Ea*, *Cpr76Bd*, *Cpr97Eb*, *Sox14*, *Cpr50Cb*, *Cad99C*, *trn*, *slow*, *moody*, *Ptp10D*, *aos*, *Cpr64Aa*, *Cpr47Ef*, *CG10641*, *CG15515*, *Cpr64Ac*, *sv*, *cue*, *CG10702*, *Pvr*, *ken*, *CG9509*, *resilin*, *lz*, *vn*, *rdo*, *CG34375*, *CG9850*, *pip*, *CG17111*, *Cpr92F*, *hbs*, *Cht7*, *Pu*, *CG34461*, *Irk2*, *Fas3*, *Cpr11A*, *CG16857*, *CG13183*, *CG13188*, *conv*, *CG16884*, *Ets98B*, *M6*, *Sesn*, *obst*-*A*, *Tsp*, *Cad96Ca*, *ft*, *nrv2*
GO:0032501	Multicellular organismal process	2.43E−06	0.000698	1.52	*bmm*, *Inx2*, *e*, *NLaz*, *Mdr65*, *CG10226*, *sas*, *fz*, *Mmp1*, *dlp*, *Cht5*, *cv*-*2*, *Oamb*, *aret*, *W*, *N*, *fng*, *CG34371*, *pk*, *Cry*, *Phk*-*3*, *CG30427*, *scb*, *Aph*-*4*, *knk*, *CG4221*, *Cht6*, *CG10936*, *Cpr49Ac*, *CG10407*, *ec*, *how*, *ds*, *ogre*, *CG5541*, *Sema*-*5c*, *CG14457*, *Fas2*, *grh*, *esg*, *miple2*, *ltd*, *CG5867*, *CG8483*, *Cg25C*, *fj*, *drd*, *Ccp84Ad*, *bnb*, *Obp56e*, *Jhe*, *kkv*, *Cpr73D*, *Dl*, *aay*, *CG12344*, *pio*, *ple*, *Cht2*, *dp*, *pot*, *Swim*, *verm*, *CG10383*, *Oatp58Dc*, *CG42326*, *pbl*, *Ser*, *Tie*, *mys*, *Sox14*, *slow*, *moody*, *Ptp10D*, *CG14259*, *aos*, *Obp83g*, *ImpL2*, *cue*, *CG2121*, *Pvr*, *CG10702*, *ken*, *lz*, *vn*, *CG34375*, *CG15117*, *pip*, *GlyP*, *Cht7*, *Pu*, *Fas3*, *Peritrophin*-*A*, *cv*, *CG2650*, *Sesn*, *CG17974*, *Tsp*, *Cad96Ca*, *ft*, *CG31189*, *nrv2*, *CG11852*
GO:0044550	Secondary metabolite biosynthetic process	5.67E−05	0.0107	5.6	*bond*, *yellow*-*h*, *yellow*-*e*, *e*, *yellow*-*d2*, *ltd*, *yellow*-*c*, *CG31121*
GO:0000003	Reproduction	4.59E−05	0.00889	3.11	*Ccp84Ad*, *NLaz*, *Peritrophin*-*A*, *CG2650*, *Aph*-*4*, *CG14259*, *Obp56e*, *CG42326*, *CG14457*, *CG15117*, *CG17974*, *CG31189*, *CG10407*, *CG8483*, *CG5867*, *CG11852*
GO:0007185	Transmembrane receptor protein tyrosine phosphatase signaling pathway	0.000412	0.0462	15.76	*CG13183*, *CG13188*, *Gp150*
GO:1901071	Glucosamine-containing compound metabolic process	1.75E−14	2.09E−11	7.58	*CG13643*, *CG13183*, *CG8192*, *Cda4*, *CG13188*, *CG13676*, *CG14304*, *Peritrophin*-*A*, *serp*, *obst*-*B*, *verm*, *CG14608*, *obst*-*A*, *Cda5*, *knk*, *Cht5*, *Cht6*, *kkv*, *Gasp*, *CG7714*, *Cht7*, *Cht2*
GO:0006030	Chitin metabolic process	8.39E−16	2.01E−12	8.56	*CG13643*, *CG13183*, *CG8192*, *Cda4*, *CG13676*, *CG13188*, *CG14304*, *Peritrophin*-*A*, *serp*, *obst*-*B*, *verm*, *CG14608*, *obst*-*A*, *Cda5*, *knk*, *Cht5*, *Cht6*, *kkv*, *Gasp*, *CG7714*, *Cht7*, *Cht2*
GO:0017144	Drug metabolic process	7.05E−09	3.61E−06	3.35	*CG13643*, *CG8192*, *Cda4*, *e*, *Duox*, *obst*-*B*, *serp*, *verm*, *CG14608*, *Cda5*, *Cht5*, *Gasp*, *Cht7*, *Pu*, *CG13183*, *CG13676*, *CG13188*, *CG14304*, *CG7059*, *Peritrophin*-*A*, *Ahcy89E*, *obst*-*A*, *knk*, *Cht6*, *kkv*, *CG7714*, *su(r)*, *Cht2*, *ple*
GO:0006022	Aminoglycan metabolic process	1.10E−12	1.13E−09	6.04	*CG3038*, *CG13643*, *CG13183*, *CG8192*, *Cda4*, *CG13676*, *CG13188*, *CG14304*, *Peritrophin*-*A*, *serp*, *obst*-*B*, *verm*, *CG14608*, *obst*-*A*, *Cda5*, *knk*, *Cht5*, *Cht6*, *kkv*, *Gasp*, *CG7714*, *Cht7*, *Cht2*
GO:0048856	Anatomical structure development	3.95E−15	7.08E−12	1.99	*CG9634*, *Mdr65*, *fz*, *sas*, *Mmp1*, *dlp*, *Cht5*, *Acp65Aa*, *aret*, *W*, *N*, *fng*, *Sb*, *spz5*, *Cry*, *vkg*, *l(3)mbn*, *Phk*-*3*, *scb*, *Aph*-*4*, *Cpr66D*, *knk*, *Cpr100A*, *Pkg21D*, *Cpr49Ac*, *Cpr49Ah*, *Cpr49Af*, *Cpr49Ae*, *how*, *Sema*-*5c*, *LanB1*, *Fas2*, *grh*, *TwdlT*, *stl*, *out*, *esg*, *Cpr47Ea*, *melt*, *DAAM*, *Cg25C*, *drd*, *Ccp84Ad*, *Ccp84Ab*, *bnb*, *spz3*, *TwdlE*, *Ccp84Aa*, *Cpr62Bc*, *Cpr62Bb*, *kkv*, *Cpr73D*, *Dl*, *aay*, *prc*, *pio*, *Cht2*, *ple*, *d*, *dp*, *CG10348*, *fw*, *pot*, *Duox*, *wdp*, *Gp150*, *serp*, *verm*, *pbl*, *Ser*, *Gasp*, *Sobp*, *Tie*, *mys*, *laccase2*, *Cpr97Ea*, *Cpr76Bd*, *Sox14*, *Cpr97Eb*, *Cpr50Cb*, *slow*, *moody*, *Ptp10D*, *Cpr64Aa*, *aos*, *Cpr47Ef*, *Cpr64Ac*, *CG15515*, *CG10641*, *sv*, *cue*, *Pvr*, *CG10702*, *ken*, *CG9509*, *resilin*, *lz*, *vn*, *CG34375*, *rdo*, *CG9850*, *pip*, *Cpr92F*, *Cht7*, *Pu*, *Irk2*, *CG34461*, *CG16857*, *Cpr11A*, *Fas3*, *conv*, *CG16884*, *M6*, *obst*-*A*, *Sesn*, *Tsp*, *Cad96Ca*, *ft*, *nrv2*
GO:0009611	Response to wounding	0.000369	0.0427	3.35	*Cht5*, *kkv*, *Spn28Dc*, *Cad96Ca*, *scb*, *Cht7*, *CG11089*, *Mmp1*, *lz*, *Cht2*, *ple*
GO:0006032	Chitin catabolic process	7.01E−07	0.000239	9.34	*Cht6*, *Cht5*, *Cda4*, *serp*, *verm*, *Cht7*, *Cda5*, *Cht2*
GO:0042737	Drug catabolic process	0.000365	0.0429	3.94	*Cht6*, *Cht5*, *Cda4*, *serp*, *verm*, *su(r)*, *Cht7*, *Cda5*, *Cht2*
GO:0022404	Molting cycle process	4.33E−06	0.00107	7.64	*Cht6*, *Cht5*, *dp*, *pot*, *e*, *Cht7*, *Cht2*, *pio*
GO:0009886	Post-embryonic animal morphogenesis	0.000163	0.0212	2.03	*d*, *dp*, *how*, *fw*, *pot*, *ds*, *Duox*, *fz*, *Mmp1*, *dlp*, *vn*, *Ser*, *Fas2*, *cv*-*2*, *rpr*, *scaf*, *mys*, *Cg25C*, *W*, *fng*, *N*, *fj*, *pk*, *trn*, *aos*, *RhoGAP15B*, *Pvr*, *Dl*, *ft*, *pio*
GO:0046667	Compound eye retinal cell programmed cell death	0.000843	0.0851	8.41	*Dl*, *W*, *N*, *ec*
GO:0060541	Respiratory system development	3.22E−05	0.00641	3.2	*dp*, *conv*, *serp*, *Ptp10D*, *verm*, *Mmp1*, *knk*, *kkv*, *grh*, *esg*, *Dl*, *nrv2*, *DAAM*, *W*, *N*, *pio*
GO:0007475	Apposition of dorsal and ventral imaginal disc-derived wing surfaces	0.000178	0.0224	6.64	*dp*, *how*, *pot*, *Dl*, *mys*, *pio*
GO:0048731	System development	0.000398	0.0453	2.08	*dp*, *ken*, *serp*, *verm*, *Mmp1*, *pbl*, *vn*, *grh*, *esg*, *mys*, *melt*, *DAAM*, *W*, *N*, *spz5*, *conv*, *spz3*, *Aph*-*4*, *Ptp10D*, *knk*, *kkv*, *Dl*, *aay*, *nrv2*, *pio*
GO:0008362	Chitin-based embryonic cuticle biosynthetic process	3.77E−08	1.42E−05	10.51	*dp*, *kkv*, *pot*, *Gasp*, *grh*, *obst*-*A*, *knk*, *Cht2*, *pio*
GO:0042335	Cuticle development	1.35E−30	4.83E−27	8.56	*dp*, *pot*, *Duox*, *resilin*, *Cht5*, *Gasp*, *grh*, *TwdlT*, *Cpr92F*, *Cpr47Ea*, *Acp65Aa*, *Cht7*, *Pu*, *laccase2*, *Cpr97Ea*, *Ccp84Ad*, *drd*, *CG34461*, *Cpr76Bd*, *Cpr97Eb*, *Cpr11A*, *Ccp84Ab*, *Cpr50Cb*, *l(3)mbn*, *TwdlE*, *Cpr64Aa*, *Cpr66D*, *Cpr62Bc*, *Ccp84Aa*, *Cpr47Ef*, *obst*-*A*, *Cpr64Ac*, *CG15515*, *knk*, *Cpr62Bb*, *kkv*, *Cpr100A*, *Cpr73D*, *Cpr49Ac*, *Cpr49Ah*, *Cpr49Af*, *Cpr49Ae*, *Cht2*, *pio*
GO:0040005	Chitin-based cuticle attachment to epithelium	0.000107	0.0167	21.02	*dp*, *pot*, *pio*
GO:0016339	Calcium-dependent cell–cell adhesion via plasma membrane cell adhesion molecules	1.16E−06	0.000379	8.85	*ds*, *Cad99C*, *Cad87A*, *Cad74A*, *Cad96Ca*, *ft*, *mys*, *scb*
GO:0044331	Cell–cell adhesion mediated by cadherin	0.000333	0.0398	7.51	*ds*, *Cad99C*, *Cad87A*, *Cad74A*, *ft*
GO:0007156	Homophilic cell adhesion via plasma membrane adhesion molecules	7.02E−09	3.87E−06	8.14	*Fas2*, *CG16857*, *Fas3*, *fw*, *ds*, *Cad99C*, *Cad96Ca*, *Cad87A*, *Cad74A*, *ft*, *fz*, *hbs*
GO:0035112	Genitalia morphogenesis	0.000178	0.0228	6.64	*Fas2*, *Pvr*, *rpr*, *scaf*, *mys*, *N*
GO:0007157	Heterophilic cell–cell adhesion via plasma membrane cell adhesion molecules	0.000119	0.0178	5.88	*Fas3*, *ds*, *ft*, *scb*, *mys*, *hbs*, *N*
GO:0042067	Establishment of ommatidial planar polarity	2.53E−08	1.01E−05	8.26	*fj*, *d*, *pk*, *fw*, *ds*, *Dl*, *ft*, *fz*, *hbs*, *aos*, *N*
GO:0090066	Regulation of anatomical structure size	0.000323	0.0393	2.23	*fj*, *dp*, *ds*, *Cad99C*, *conv*, *slow*, *fz*, *serp*, *verm*, *Mmp1*, *knk*, *obst*-*A*, *pbl*, *Fas2*, *kkv*, *grh*, *Gasp*, *Cad96Ca*, *ft*, *nrv2*, *DAAM*, *aret*
GO:0035150	Regulation of tube size	7.28E−09	3.48E−06	6.18	*fj*, *ds*, *conv*, *fz*, *serp*, *verm*, *Mmp1*, *knk*, *obst*-*A*, *Fas2*, *kkv*, *Gasp*, *grh*, *ft*, *nrv2*
GO:0035159	Regulation of tube length, open tracheal system	7.94E−10	5.18E−07	8.54	*fj*, *ds*, *conv*, *serp*, *fz*, *verm*, *Mmp1*, *knk*, *Fas2*, *kkv*, *grh*, *ft*, *nrv2*
GO:0035152	Regulation of tube architecture, open tracheal system	1.29E−08	5.42E−06	4.91	*fj*, *ds*, *conv*, *serp*, *fz*, *verm*, *Mmp1*, *obst*-*A*, *knk*, *Fas2*, *kkv*, *uif*, *Gasp*, *grh*, *ft*, *mys*, *nrv2*, *DAAM*
GO:0098742	Cell–cell adhesion via plasma-membrane adhesion molecules	9.80E−09	4.39E−06	6.06	*fw*, *CG16857*, *Fas3*, *ds*, *Cad99C*, *fz*, *scb*, *Fas2*, *Cad96Ca*, *Cad74A*, *Cad87A*, *ft*, *mys*, *hbs*, *N*
GO:0098609	Cell–cell adhesion	2.59E−06	0.000714	4.09	*fw*, *Fas3*, *CG16857*, *ds*, *Cad99C*, *fz*, *scb*, *Fas2*, *Cad87A*, *Cad74A*, *Cad96Ca*, *ft*, *mys*, *hbs*, *N*
GO:0007155	Cell adhesion	4.01E−11	3.19E−08	4.26	*fw*, *how*, *ds*, *Swim*, *fz*, *Mmp1*, *sprt*, *LanB1*, *Fas2*, *Cad74A*, *mys*, *hbs*, *N*, *Fas3*, *CG16857*, *zye*, *Cad99C*, *trn*, *scb*, *CG15080*, *ImpL2*, *Tsp*, *Cad87A*, *Cad96Ca*, *ft*, *Dl*, *nrv2*, *prc*
GO:0090099	Negative regulation of decapentaplegic signaling pathway	0.000995	0.0964	12.61	*Irk2*, *Fs*, *scaf*
GO:0006031	Chitin biosynthetic process	7.01E−05	0.0129	14.01	*kkv*, *CG13183*, *CG13188*, *knk*
GO:0060439	Trachea morphogenesis	0.000157	0.0209	12.01	*kkv*, *CG13183*, *CG13188*, *verm*
GO:0048085	Adult chitin-containing cuticle pigmentation	0.000722	0.0773	5.25	*kkv*, *e*, *Duox*, *CG10625*, *CG9134*, *ple*
GO:0001838	Embryonic epithelial tube formation	0.000995	0.0977	12.61	*kkv*, *Mmp1*, *knk*
GO:0048585	Negative regulation of response to stimulus	0.00095	0.0946	1.87	*nimA*, *slif*, *wdp*, *fz*, *dlp*, *pbl*, *l(2)34Fc*, *Fas2*, *Ser*, *uif*, *Coop*, *Tie*, *scaf*, *GlyP*, *fng*, *N*, *Irk2*, *pk*, *Spn28Dc*, *aos*, *Sesn*, *ImpL2*, *CG4096*, *Fs*, *Cad96Ca*, *CG10702*, *Pvr*, *ft*
GO:0023057	Negative regulation of signaling	0.000788	0.0819	1.92	*nimA*, *wdp*, *fz*, *dlp*, *pbl*, *Fas2*, *Ser*, *uif*, *Coop*, *Tie*, *scaf*, *fng*, *N*, *Irk2*, *pk*, *CG8317*, *aos*, *Sesn*, *ImpL2*, *CG4096*, *Fs*, *Cad96Ca*, *CG10702*, *Pvr*, *ft*, *egr*, *CG12344*
GO:0048067	Cuticle pigmentation	1.84E−07	6.59E−05	7.01	*Pu*, *kkv*, *yellow*-*h*, *yellow*-*e*, *e*, *Duox*, *yellow*-*d2*, *CG10625*, *CG9134*, *yellow*-*c*, *ple*
GO:0043473	Pigmentation	2.65E−06	0.000705	5.04	*Pu*, *kkv*, *yellow*-*h*, *yellow*-*e*, *e*, *Duox*, *yellow*-*d2*, *ltd*, *CG10625*, *CG9134*, *yellow*-*c*, *ple*
GO:0046148	Pigment biosynthetic process	0.000283	0.0351	3.45	*Pu*, *se*, *santa*-*maria*, *yellow*-*h*, *yellow*-*e*, *e*, *yellow*-*d2*, *ltd*, *yellow*-*c*, *DhpD*, *CG31121*
GO:0007508	Larval heart development	0.000107	0.0163	21.02	*scb*, *mys*, *prc*
GO:0035001	Dorsal trunk growth, open tracheal system	0.000157	0.0213	12.01	*scb*, *mys*, *verm*, *Mmp1*
GO:0035161	Imaginal disc lineage restriction	0.000843	0.0863	8.41	*Ser*, *Dl*, *fng*, *N*
GO:0007451	Dorsal/ventral lineage restriction, imaginal disc	7.01E−05	0.012	14.01	*Ser*, *Dl*, *N*, *fng*
GO:0035170	Lymph gland crystal cell differentiation	0.000412	0.0455	15.76	*Ser*, *lz*, *N*
GO:0042438	Melanin biosynthetic process	1.18E−05	0.00282	13.14	*yellow*-*h*, *yellow*-*e*, *e*, *yellow*-*d2*, *yellow*-*c*

### Transcription factor-binding motifs are enriched in the promoters of blue light-regulated genes

What factors mediate the blue light-induced changes in gene expression in photoreceptors? Our qPCR analysis indicated that there were different pathways associated with blue light-upregulated and downregulated changes in gene expression. An intact phototransduction pathway and calcium influx were only required for upregulation, but not downregulation, of genes in response to blue light. Thus, these data suggest that light-induced calcium influx activates the blue light-upregulated genes, whereas the blue light-downregulated genes are repressed, perhaps transiently, by exposure to light itself. To identify potential transcription factors that could mediate blue light-induced changes in gene expression, we examined the promoters of blue light up- or downregulated genes for enriched sequence motifs using hypergeometric optimization of motif enrichment (HOMER) [32]. Using this approach, we identified different sets of significantly enriched promoter motifs for blue light up- and downregulated genes (Additional file 1: Fig. S4, Fig. S5). These promoter motifs corresponded to potential binding sites for different transcription factors (Additional file 4: Table S3). Four of the promoter motifs identified for the blue light-upregulated genes contained potential binding sites for Heat shock factor (Hsf), a key mediator of the stress response [33]. In addition, a potential binding site for the AP-1 transcription factor, composed of Jun-related antigen (Jra) and Kayak (Kay) in flies, was present in one of the promoter motifs identified for the blue light-upregulated genes. Interestingly, a transcription co-activator that is important for redox-sensing by AP-1, *multiprotein bridging factor 1* (*mbf1*), was upregulated in response to blue light [34]. Surprisingly, while expression of the unfolded protein response mediator *Inositol*-*requiring enzyme*-*1* (*Ire1*) was upregulated in response to blue light, we only identified one potential binding site for the Ire1-activated transcription factor, X box binding protein-1 (Xbp1), in the blue light-downregulated genes. One attractive candidate for a transcription factor that could mediate the light and calcium-dependent changes in gene expression is the Calmodulin-binding transcription activator (Camta) that activates expression of genes that are involved in deactivation of rhodopsin signaling [35]. *Camta* expression was reduced upon blue light exposure, and a potential Camta binding site (CGCG motif, motif 28) was present in the promoters of blue light-upregulated genes (Additional file 1: Fig. S4). However, canonical Camta-target genes such as *F box and leucine*-*rich*-*repeat gene 4* (*Fbxl4*) and *CG7227* were not differentially expressed in response to blue light, suggesting that these Camta-regulated genes do not respond to blue light under the conditions used for our experiment.

## Discussion

**Fig. 6:**
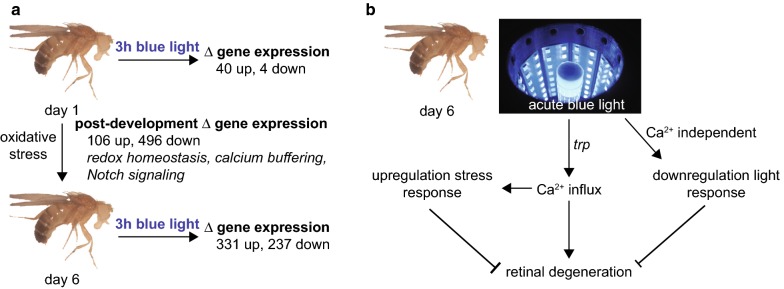
Blue light induces neuroprotective gene expression changes in photoreceptors via calcium-dependent and independent pathways. **a** Newly-eclosed (day one) flies express high levels of genes that enable them to withstand blue light exposure. Exposure to standard white light conditions during the first week of life increases oxidative stress levels in photoreceptors, correlating with increased expression of some stress response genes. Concomitantly, post-development transitions in gene expression between newly-eclosed and mature flies result in reduced levels of genes required to maintain redox homeostasis and buffer calcium. Following exposure to acute blue light, mature six-day-old flies activate a strong neuroprotective gene expression program in an effort to prevent retinal degeneration. **b** Blue light-induced changes in gene expression in six-day-old flies include calcium-dependent upregulation of stress response genes, and calcium-independent downregulation of genes involved in light response such as calcium and ion channels. This gene expression program enables six-day-old flies to resist moderate (3 h) blue light exposure, but is not sufficient to prevent retinal degeneration when flies are subjected to longer periods of blue light (8 h)

The eye is susceptible to light-induced oxidative stress, which has been implicated in photoreceptor damage in a variety of eye diseases [36, 37]. To characterize the light stress response in *Drosophila* photoreceptors, we profiled the transcriptome of photoreceptors exposed to high intensities of blue light. Although longer durations of blue light induce severe retinal degeneration in white-eyed flies [19, 38], shorter exposures to blue light induced major gene expression changes in photoreceptors but did not cause retinal degeneration. Instead, blue light induced expression of a broad range of genes involved in stress response, together with a concomitant reduction in expression of genes required for the light response including voltage-gated calcium, potassium and chloride ion channels. We expect that these transcriptional changes would result in altered protein levels; however, this has not been tested in this study. Previous studies showed that very young flies (1 day post-eclosion) were resistant to blue light-induced retinal degeneration, and our work revealed that the blue light-induced transcriptional changes differed according to the age of the fly; mature flies (6 days post-eclosion) showed substantially more differentially expressed genes in response to blue light exposure than very young flies (1 day post-eclosion). The increase in susceptibility to blue light between day one and six correlated with developmental transitions in photoreceptor gene expression, which included reduced expression of genes that function in redox and calcium homeostasis (Fig. 6a). Together, our data support a model in which mature adult flies upregulate stress response pathways in an effort to deal with light-induced oxidative stress, and concomitantly quench the light response to diminish phototransduction-associated calcium influx (Fig. 6b). Newly-eclosed flies might be able to withstand blue light exposure better because of an increased capacity to buffer the calcium influx and oxidative stress resulting from prolonged phototransduction. Indeed, relatively young, yet mature, flies (day six) can withstand moderate blue light exposure without significant retinal degeneration but lose the ability to resist longer durations of light exposure. Recent work demonstrated that white-eyed flies (*w*^*1118*^), but not their pigmented counterparts, undergo age-associated retinal degeneration under normal light/dark cycles by 30 days [39]. Thus, the acute blue light paradigm used in our study may reveal insight into mechanisms associated with age-associated retinal degeneration.

The transient, blue light-dependent downregulation of the calcium channel gene, *trp*, in day six flies corresponds well with our previous observations that mutations in *trp* suppress blue light-induced retinal degeneration. However, many voltage-gated potassium and chloride channels were also downregulated in response to blue light. Could decreasing activity of potassium or chloride channels ameliorate phototoxicity in flies? Excessive calcium influx is associated with brain ischemia-induced neuronal death, and potassium channel blockers reduced hypoxia-induced neuronal apoptosis in rodent models of ischemia [40]. However, eye-specific knockdown of *ATPα*, a subunit of a sodium/potassium channel, using the *longGMR*-*Gal4* driver caused age-dependent retinal degeneration in flies [41]. It is currently unclear whether transient repression of other voltage-gated ion channels in photoreceptors could attenuate retinal degeneration under phototoxic conditions.

How could exposure to blue light downregulate expression of genes, independent of phototransduction or calcium influx? In *Drosophila*, the blue light receptor cryptochrome (*cry*) entrains circadian rhythms to light–dark cycles via light-activated degradation of the clock protein Timeless (*tim*) [42]. Fly photoreceptors possess a functional circadian clock and express *PAR*-*domain protein 1* (*Pdp1*), *tim*, and *cry* [43–45]. We observed an enrichment of genes involved in circadian rhythm among the blue light-downregulated genes (Table 2). Regulators of the circadian clock including *tim*, *Pdp1*, and *vrille* (*vri*) were downregulated in response to blue light in day six, but not day one flies (Additional file 2: Table S1). When we compared the blue light-regulated genes in six-day-old flies with genes showing rhythmic expression patterns in fly heads [46], we found that 14 and 24 of the blue light up- and downregulated genes respectively (including *trp*) overlapped with the 331 genes showing rhythmic expression profiles in heads. While in flies Cry is thought to mainly function by mediating light-dependent degradation of Timeless, some data suggest that Cry also acts as a transcriptional repressor in peripheral circadian clocks because loss of *cry* and *period (per*) in the eye leads to ectopic expression of *tim* [47]. However, we would expect to observe increased, rather than decreased, *tim* levels following blue light exposure if Cry-mediated transcriptional repression was involved because blue light causes degradation of Cry [42]. Thus, we propose that some unknown part of the circadian gene regulatory machinery regulates a light-dependent gene expression program in photoreceptors that attenuates the light response under strong illumination. Other transcription factors such as Kayak, which has a promoter motif in the blue light-upregulated genes, have been shown to affect expression of circadian-regulated genes in pacemaker neurons [48]. We note that the design of our study presents some difficulty in teasing out a potential role for circadian pathway components because we cannot readily distinguish between gene expression changes that occur in response to blue light and expression changes that occur in response to dark incubation, which we used as a control for these experiments. Our data suggest that the dark incubation does not itself cause major changes in gene expression because day one flies showed very few gene expression changes in response to blue light relative to dark control. Further, the subsets of genes tested by qPCR in dissected eyes showed similar directions of change to the RNA-seq analysis when normalized to a pre-treatment sample (Fig. 3). Thus, we speculate that some components of the circadian machinery are coopted in *Drosophila* photoreceptors to repress the expression of light response pathway genes in response to strong illumination.

## Conclusions

Although light is essential for vision, it also poses a stress to photoreceptor cells within the eye. Young flies at 6 days post-eclosion undergo retinal degeneration when exposed to prolonged blue light exposure. Here, we show that exposure to blue light induces substantial gene expression changes in photoreceptors from six-day-old flies. In these flies, blue light upregulates stress response pathways and downregulates light response genes to mitigate oxidative stress, and quench the light response. Newly-eclosed flies, which are resilient to blue light-induced retinal degeneration, show no such changes in gene expression. Our data suggest that newly-eclosed flies express higher levels of genes that help withstand light stress because of their recent transition from the developing pupal to early adult stage. Together, the results from this study provide insight into neuroprotective pathways utilized by photoreceptors to resist light-induced oxidative stress.

## Methods

### Stocks, genetics, and blue light treatment

All genotypes used in this study are described in Additional file 3: Table S4. Mated male flies were used for all experiments. Flies were cultured on standard cornmeal food at 25 °C with 12 h/12 h light/dark cycle except for *ninaE*^*7*^ and *trp*^*9*^ flies, which together with the *w*^*1118*^ controls for those experiments, were raised in the dark prior to blue light treatment to prevent light-dependent retinal degeneration [49]. Flies homozygous for KASH-GFP, *P*{*w*^+*mC*^= *UAS*-*GFP*-*Msp300KASH*}*attP2*, under the control of Rh1-Gal4 (*P*{*ry*^+*t7.2*^= *rh1*-*GAL4*}*3*, *ry*^*506*^ [BL8691] were crossed to *cn bw* to deplete eye pigments [22]. For aging experiments, 400 male flies were collected from 0 to 8 h post-eclosion and aged for 12 h (day one; 12–19 h) or 6 days. Flies were exposed to 3 h of blue light (λ = 465 nm) at 8000 lx (2 mW/cm^2^) using a custom designed optical stimulator with temperature control (23–25 °C) [38].

### Immunostaining and confocal microscopy

Adult fly retinas were dissected and stained with phalloidin (A22287, 1:100, Thermo Fisher Scientific) as described previously [20]. Laser scanning confocal imaging was performed using a Nikon A1R inverted confocal microscope under a 60X/1.30 NA oil immersion Nikon Plan Fluor objective. Confocal images were collected either as single planes or 1.0 μm z-stacks using NIS-Elements software. Retinal cell degeneration was quantified by assessing rhabdomere loss (presence/absence phalloidin-positive rhabdomere) for R1–R6 cells per ommatidium using stacked images. Rhabdomere loss was quantified in five independent male flies (single eye/fly) for four independent light exposures (paired blue light versus dark controls).

### RNA isolation, RNA-seq, and qPCR analysis

RNA-seq analysis: Heads were collected from ~ 400 male flies of the indicated treatments and ages and GFP-labeled photoreceptor nuclei were affinity purified as previously described [20, 21]. Total nuclear RNA was extracted using Trizol reagent (Life Technologies), followed by Direct-zol RNA Micro-prep kit (R2062, Zymo Research) including DNase treatment. RNA (35 ng) was used to generate uniquely barcoded, strand-specific and rRNA depleted library using NuGen Ovation RNA seq Systems 1-16 for Model Organism (0350, Nugen). All samples were added to a single pool that was clustered in two lanes of a HiSeq 2500 single-end rapid flowcell to generate 50 base reads per cluster. Quantitative PCR (qPCR) analysis: RNA was isolated from dissected eyes using Trizol (Invitrogen) and qPCR analysis was performed on cDNA generated from 100 ng RNA using random hexamers relative to a standard curve of serially diluted cDNA. Relative expression for each gene was normalized to the geometric mean of two reference genes (*eukaryotic translation initiation factor 1A*, *eIF1A* and *Ribosomal protein L32*, *RpL32*). Primers are listed in Additional file 4: Table S5.

### RNA-seq data analysis

Three biological samples were analyzed for each of the following ages and treatments: day one 3 h dark (pre-isolation, whole head homogenate), day one 3 h dark (post-isolation), day one 3 h blue (post-isolation), day six 3 h dark (post-isolation), day six 3 h blue (post-isolation). Reads were trimmed using Trimmomatic (v0.36) and mapped against the bowtie2 (v2.3.2) [50] indexed *D. melanogaster* genome (Drosophila_melanogaster.BDGP6.89) using Tophat (v 2.1.1) [51]. The raw counts matrix was generated by Htseq-count (v0.7.0) applying strand-specific assay (fr-secondstrand), union mode, and default parameters [52]. Differential expression analysis was performed on genes with greater than one count per million (CPM) in at least three samples. Differentially expressed genes were detected using *glmTreat* generalized linear model analysis in edgeR (v3.18.1) [53] with a FDR of < 0.05. A FC of 2 was applied to *glmTreat* analysis of the pre versus post samples only. Gene set enrichment analysis between age-regulated genes (day 10 vs day 40) [20] and differentially expressed genes between day one and day six (dark controls) was performed using *mroast* and visualized using *barcode plot* in edgeR. All plots were generated in R (v3.4.1) using custom scripts.

### GO term analysis

GO term enrichment analysis was performed using GOrilla [54] relative to the background gene set of all expressed genes with CPM > 1 in at least three of the samples. Only GO terms with non-redundant gene members are shown in Tables 1 and 2. Complete GO term enrichment analyses and parameters used for GOrilla are described in Additional file 3: Table S2.

### Motif analysis

Significantly-enriched promoter motifs were identified using HOMER (v4.9, Hypergeometric Optimization of Motif EnRichment) [32] as previously described [20]. The background gene set of all expressed genes with CPM > 1 in at least three of the samples was used for enrichment analysis.

## Additional files

**Additional file 1: Fig. S1.** The blue light treatment conditions used for RNA-seq analysis do not induce retinal degeneration. **Fig. S2.** Affinity-enrichment of photoreceptor nuclear RNA from day one dark-treated flies. **Fig. S3.** Newly-eclosed flies do not show any unique blue light-induced gene expression changes. **Fig. S4.** Promoter motifs enriched at blue light-regulated genes. **Fig. S5.** Distribution of promoter motifs in blue light-regulated genes.

**Additional file 2: Table** **1.** Significantly differentially expressed genes identified under each comparison.

**Additional file 3: Table** **2.** GO term analysis of differentially regulated genes.

**Additional file 4: Table** **3.** Transcription factors matches for all motifs identified for blue light-regulated genes.

**Additional file 5: Table** **4.** Fly stocks used in this study.

**Additional file 6: Table** **5.** Primers used in this study.

## Abbreviations

CPM: counts per million
FDR: false discovery rate
FC: fold change
GFP: green fluorescent protein
GEO: gene expression omnibus
GO: gene ontology
h: hour
HOMER: hypergeometric optimization of motif enrichment
KASH: Klarsicht, Anc-1, Syn3-1 homology
qPCR: quantitative polymerase chain reaction
Rh1: rhodopsin 1
Trp: transient receptor potential
