# Supplementary material for: Blue light induces a neuroprotective gene expression program in Drosophila photoreceptors
Source: BMC Neurosci. 2018 Jul 20;19:43. doi: 10.1186/s12868-018-0443-y (PMC6053765; doi:10.1186/s12868-018-0443-y)
Supplement: Supplementary file 1 — Additional file 1: Fig. S1. The blue light treatment conditions used for RNA-seq analysis do not induce retinal degeneration. Fig. S2. Affinity-enrichment of photoreceptor nuclear RNA from day one dark-treated flies. Fig. S3. Newly-eclosed flies do not show any unique blue light-induced gene expression changes. Fig. S4. Promoter motifs enriched at blue light-regulated genes. Fig. S5. Distribution of promoter motifs in blue light-regulated genes. [file 12868_2018_443_MOESM1_ESM.pdf]

SUPPLEMENTAL FIGURE LEGENDS:

Fig. S1: The blue light treatment conditions used for RNA-seq analysis do not induce retinal degeneration. (A) Confocal microscopy images of adult retinas stained with phalloidin (red) from male white-eyed *cn, bw; Rh1-Gal4, UAS-GFP-Msp300KASH* flies at one or six days post-eclosion exposed to 3h blue light or dark (control). Flies were raised in 12h/12h light/dark conditions prior to treatment. Retinas were dissected and immunostained following 12 days dark incubation post-treatment to assess rhabdomere loss. Scale bars, 20 μm. (B) Box plots showing rhabdomere loss quantified using confocal images. Means are shown by crosses. Points are overlayed for individual animals (single eye/animal) representing 4 independent light treatments with 5 flies per treatment. The distribution for each blue light-treated group was compared with the dark control for the same age using Kruskal-Wallis test. ns, not significant.

Figure S2: Affinity-enrichment of photoreceptor nuclear RNA from day one dark-treated flies. (A) Principal component analysis for pre- and post-isolation RNA-seq samples from day one male dark-treated (control) flies based on counts per million (CPM). (B) Volcano plot showing the fold change post-enrichment plotted as $\log_2$(fold change) for each gene relative to its false discovery rate ($-\log_2$[FDR]). Genes with significantly differential expression (FDR < 0.05 and FC > 2, dotted lines) are highlighted in red or blue, and GFP is shown in green for comparison.

Figure S3: Newly-eclosed flies do not show any unique blue light-induced gene expression changes. (A) Proportional Venn diagram showing the overlap between genes that are significantly differentially expressed in response to blue light in day six or day one photoreceptors. The six genes that are significantly differentially expressed only in day one photoreceptors are listed in the inset panel (red, upregulated; blue, downregulated). (B) Volcano plot showing the differential gene expression profile of the six day one-specific genes (yellow) in blue light-exposed day six photoreceptors. Fold change was plotted as $\log_2$(fold change) of a subset of genes relative to their false discovery rate ($-\log_2$[FDR]), as in Figure 2B.

Figure S4: Promoter motifs enriched at blue light-regulated genes. Significantly-enriched promoter sequence motifs identified for day six blue light down- (A) or up-regulated (B) genes. The percentage of genes in the target gene set (T:%) and *p*-value for each motif are shown

(enrichment in target versus background). Transcription factor matches corresponding to each motif are described in Table S3.

Figure S5: Distribution of promoter motifs in blue light-regulated genes. Heatmaps showing the presence of the indicated sequence motifs (columns) in the promoter of blue light-upregulated (left panel) or downregulated (right panel) genes (rows). Only genes that have at least one of the sequences motifs in their promoters are shown.

Hall_FigS1

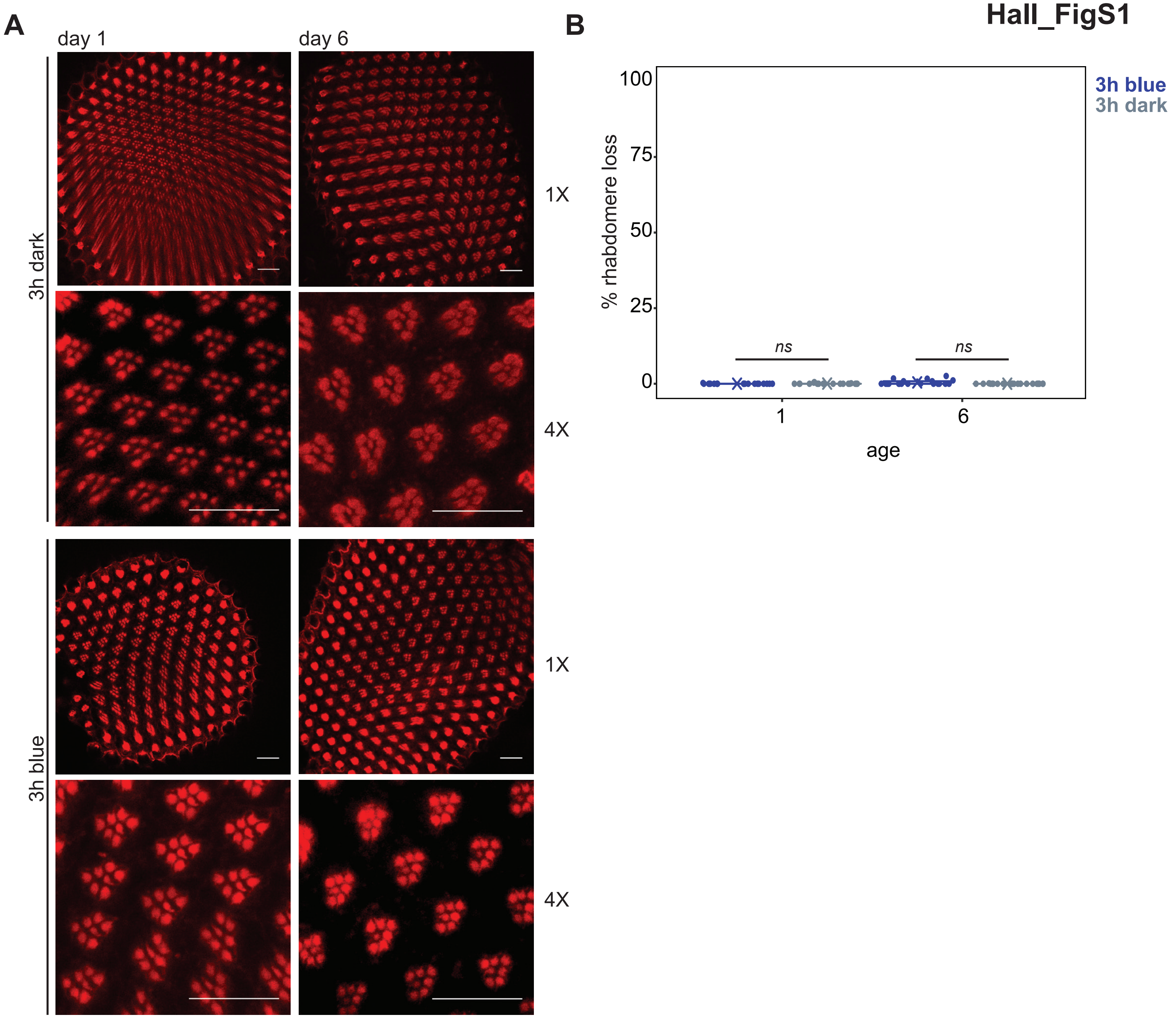
A
day 1
day 6
3h dark
3h blue
1X
4X
1X
4X
B
100
75
50
25
0
% rhabdomere loss
ns
ns
1
6
age
3h blue
3h dark

Hall_FigS2

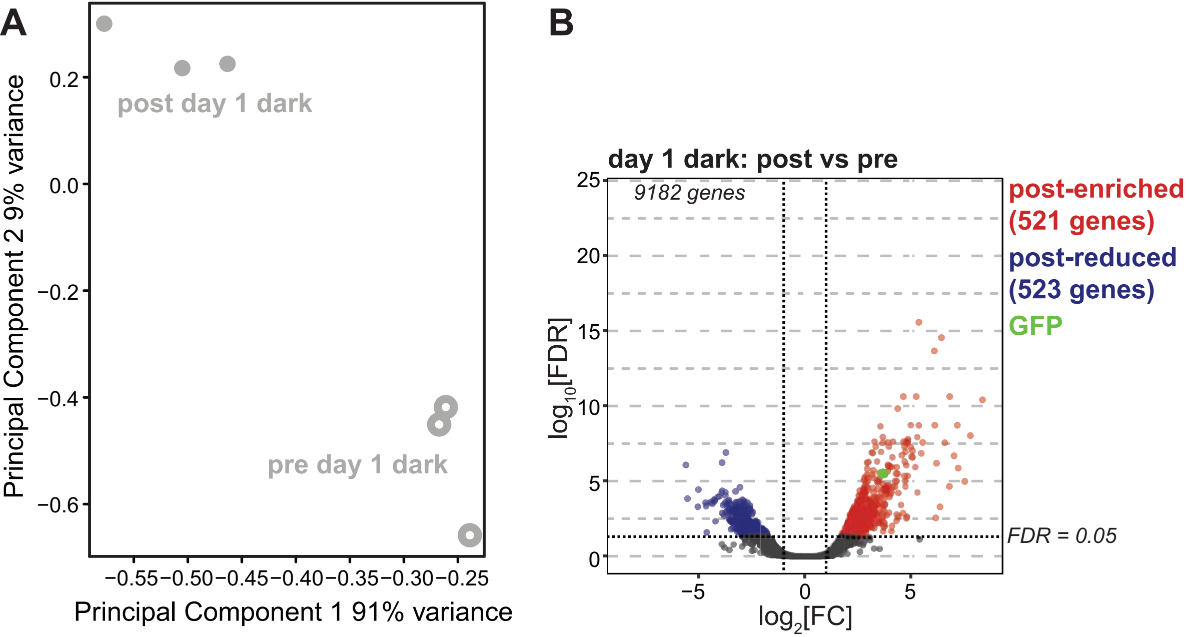

A
post day 1 dark
pre day 1 dark
0.2
0.0
−0.2
−0.4
−0.6
−0.55 −0.50 −0.45 −0.40 −0.35 −0.30 −0.25
Principal Component 2 9% variance
Principal Component 1 91% variance
B
day 1 dark: post vs pre
9182 genes
post-enriched (521 genes)
post-reduced (523 genes)
GFP
FDR = 0.05
25
20
15
10
5
0
−5 0 5
log10[FDR]
log2[FC]

Hall_FigS3

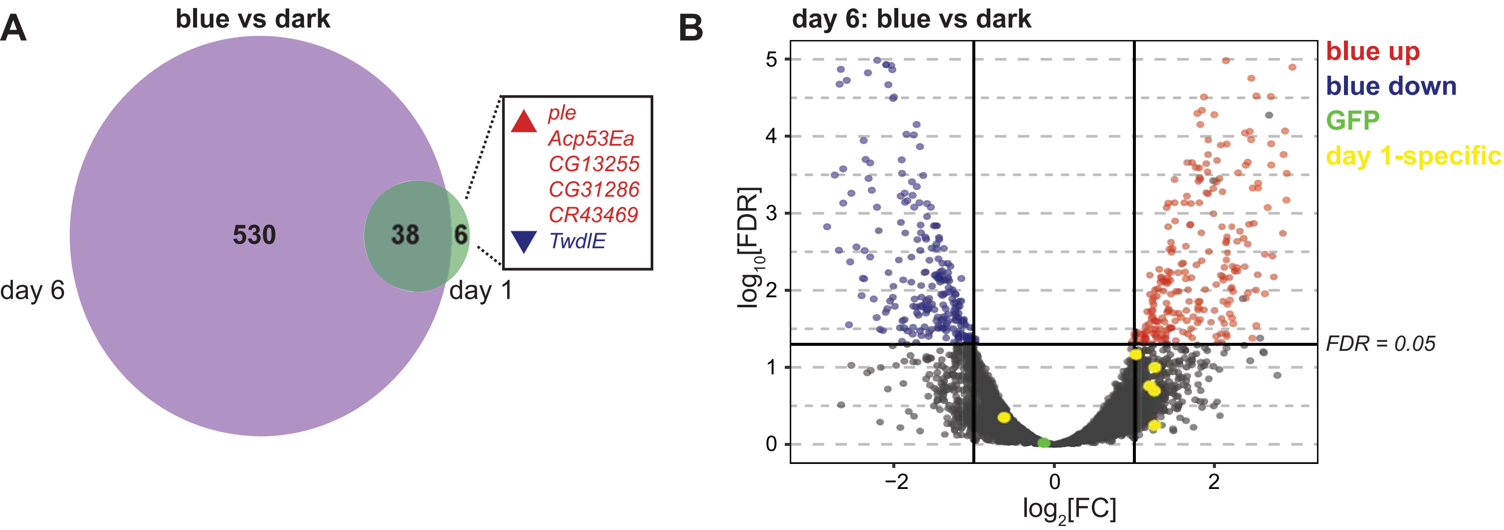
A
blue vs dark
530
38
6
day 6
day 1
ple
Acp53Ea
CG13255
CG31286
CR43469
TwdlE
B
day 6: blue vs dark
blue up
blue down
GFP
day 1-specific
FDR = 0.05
5
4
3
2
1
0
−2
0
2
log10[FDR]
log2[FC]

A day 6 blue down motifs

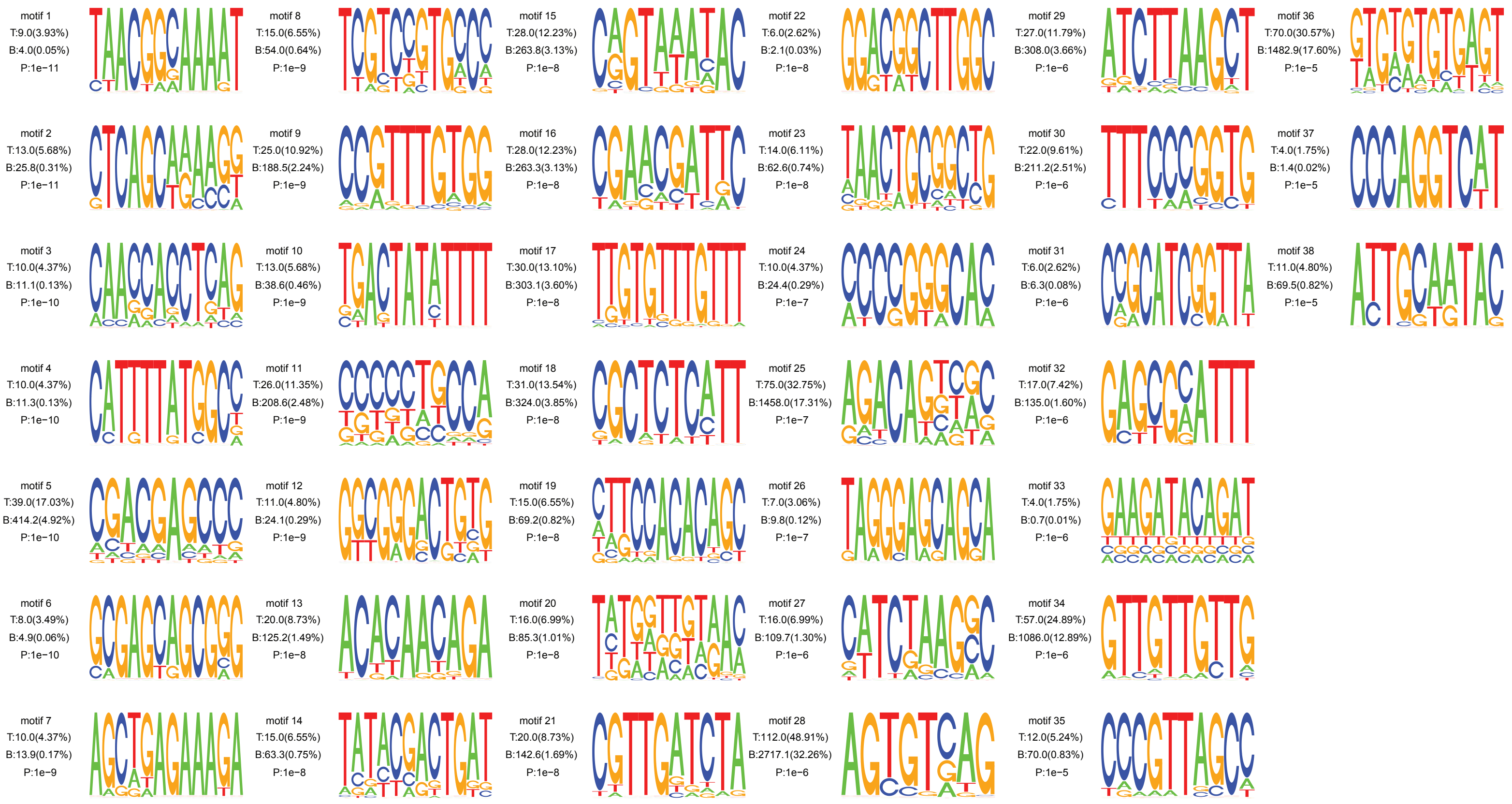

B day 6 blue up motifs

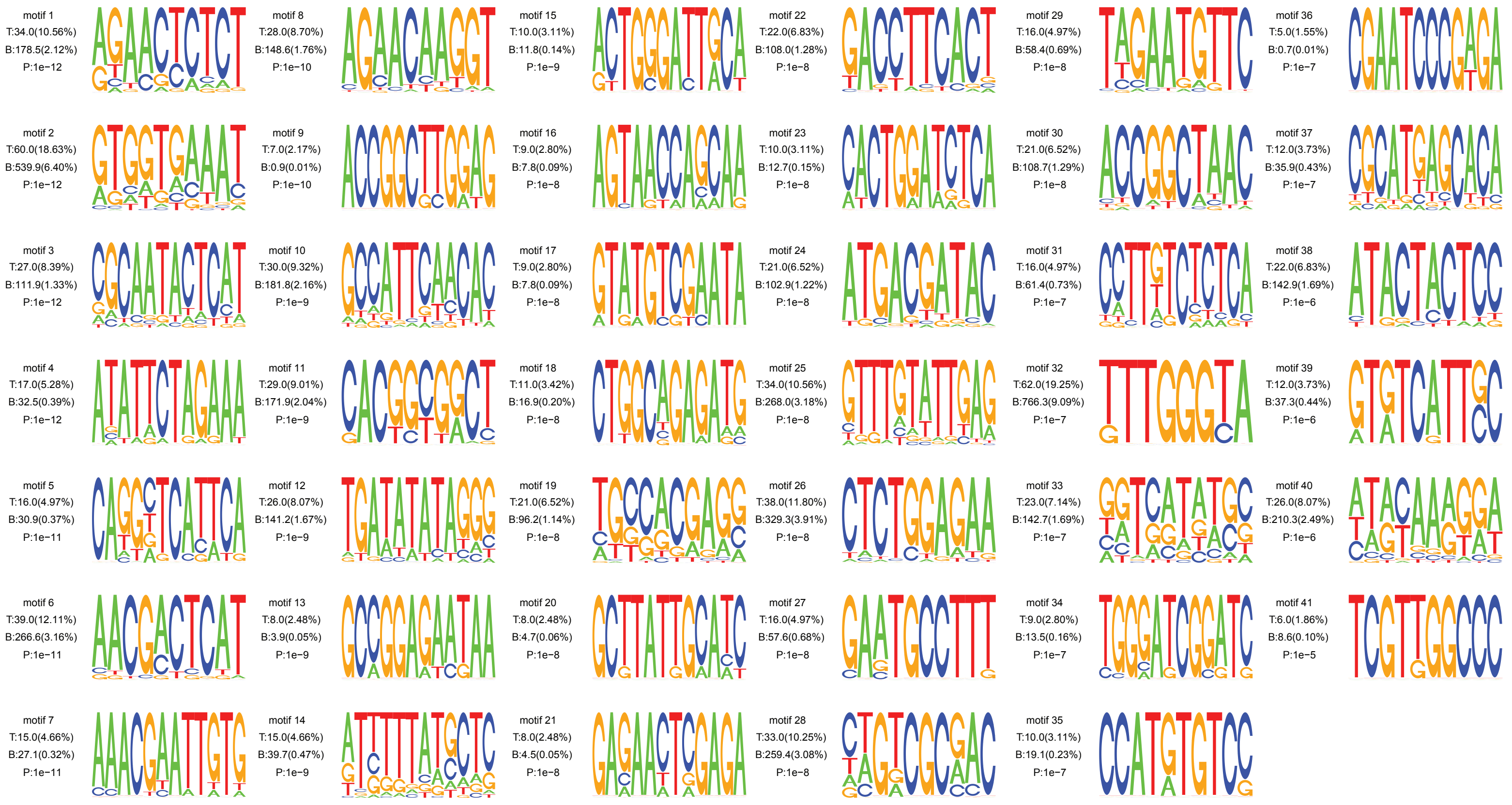

Hall_FigS5

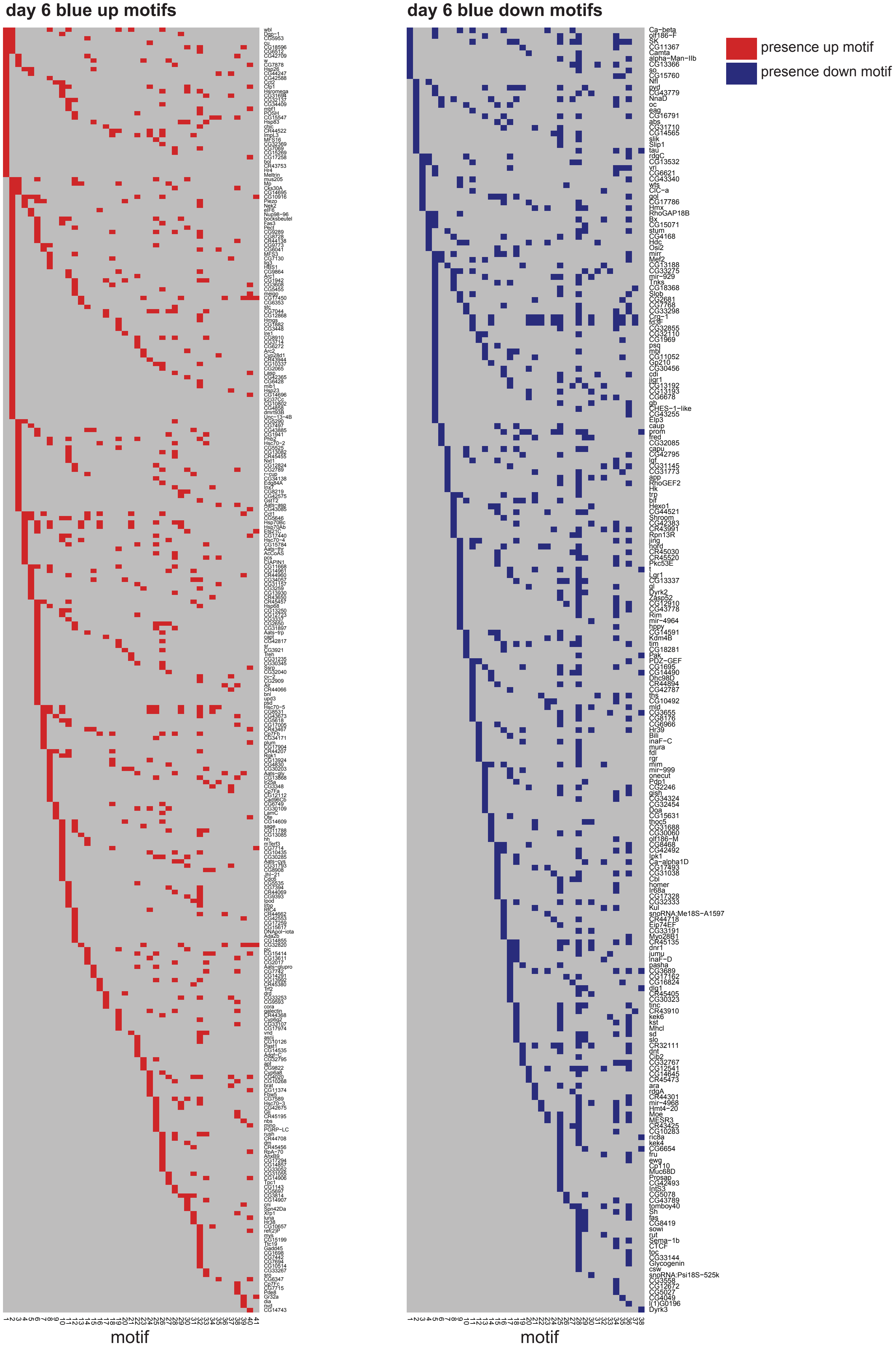
day 6 blue up motifs
day 6 blue down motifs
presence up motif
presence down motif
motif
motif
