# Supplementary material for: Blue light induces a neuroprotective gene expression program in Drosophila photoreceptors
Source: BMC Neurosci. 2018 Jul 20;19:43. doi: 10.1186/s12868-018-0443-y (PMC6053765; doi:10.1186/s12868-018-0443-y)
Supplement: Supplementary file 5 — Additional file 5: Table 4. Fly stocks used in this study. [file 12868_2018_443_MOESM5_ESM.pdf]

Fly stock	Detailed genotype	Source	Stock number	Reference
w^{1118}	w^{1118}	Bloomington	BL3605	[1]
ninaE^{7}	w^{1118};; *ninaE*^{7}	Bloomington	BL1613	[2, 3]
trp^{9}	w^{*};; *trp*^{9}	Bloomington	BL9046	[4]
cn bw	cn^{1} bw^{1}	Bloomington	BL264	[5]
Rh1-Gal4 > KASH-GFP	w^{1118};; *P{w*$^{+mC}$*=[UAS-GFP-Msp300KASH}attP2, P{ry*$^{+t7.2}$*=rh1-GAL4}3, ry*^{506}	Weake lab	NA	[6]
cn bw; Rh1-Gal4 > KASH-GFP	w^{1118}; cn^{1} bw^{1}; *P{w*$^{+mC}$*=[UAS-GFP-Msp300KASH}attP2, P{ry*$^{+t7.2}$*=rh1-GAL4}3, ry*^{506}	This study	NA	

Table S4. Fly stocks used in this study.

SUPPLEMENTAL REFERENCES FOR TABLE S4

1. Rabinow L, Birchler JA: **A dosage-sensitive modifier of retrotransposon-induced alleles of the Drosophila white locus**. *EMBO J* 1989, **8**(3):879-889.
2. Kumar JP, Ready DF: **Rhodopsin plays an essential structural role in Drosophila photoreceptor development**. *Development* 1995, **121**(12):4359-4370.
3. Kurada P, O'Tousa JE: **Retinal degeneration caused by dominant rhodopsin mutations in Drosophila**. *Neuron* 1995, **14**(3):571-579.
4. Wang T, Jiao Y, Montell C: **Dissecting independent channel and scaffolding roles of the Drosophila transient receptor potential channel**. *J Cell Biol* 2005, **171**(4):685-694.
5. Tearle R: **Tissue specific effects of ommochrome pathway mutations in Drosophila melanogaster**. *Genet Res* 1991, **57**(3):257-266.
6. Hall H, Medina P, Cooper DA, Escobedo SE, Rounds J, Brennan KJ, Vincent C, Miura P, Doerge R, Weake VM: **Transcriptome profiling of aging Drosophila photoreceptors reveals gene expression trends that correlate with visual senescence**. *BMC Genomics* 2017, **18**(1):894.
